# Gene Therapy Using Recombinant AAV Type 8 Vector Encoding TNAP‐D_10_ Improves the Skeletal Phenotypes in Murine Models of Osteomalacia

**DOI:** 10.1002/jbm4.10709

**Published:** 2022-12-15

**Authors:** Flavia Amadeu de Oliveira, Fatma F. Mohamed, Yuka Kinoshita, Sonoko Narisawa, Colin Farquharson, Koichi Miyake, Brian L Foster, Jose Luis Millan

**Affiliations:** ^1^ Human Genetics Program Sanford Burnham Prebys Medical Discovery Institute La Jolla CA USA; ^2^ Division of Biosciences, College of Dentistry The Ohio State University Columbus OH USA; ^3^ The Royal (Dick) School of Veterinary Studies (RDSVS), The Roslin Institute University of Edinburgh Edinburgh UK; ^4^ Department of Gene Therapy Nippon Medical School Tokyo Japan

**Keywords:** ANALYSIS/QUANTITATION OF BONE, ANIMAL MODELS, BONE QCT/MICRO‐CT, DENTAL BIOLOGY, DISEASES AND DISORDERS OF/RELATED TO BONE, GENETIC ANIMAL MODELS, OSTEOMALACIA AND RICKETS, PRECLINICAL STUDIES, GENE THERAPY

## Abstract

Hypophosphatasia (HPP), caused by loss‐of‐function mutations in the *ALPL* gene encoding tissue‐nonspecific alkaline phosphatase (TNAP), is characterized by skeletal and dental hypomineralization that can vary in severity from life‐threatening to milder manifestations only in adulthood. PHOSPHO1 deficiency leads to early‐onset scoliosis, osteomalacia, and fractures that mimic pseudo‐HPP. Asfotase alfa, a life‐saving enzyme replacement therapy approved for pediatric‐onset HPP, requires subcutaneous injections 3 to 6 times per week. We recently showed that a single injection of an adeno‐associated virus vector serotype 8 harboring TNAP‐D_10_ (AAV8‐TNAP‐D_10_) effectively prevented skeletal disease and prolonged life in *Alpl* ^*−/−*^ mice phenocopying infantile HPP. Here, we aimed to determine the efficacy of AAV8‐TNAP‐D_10_ in improving the skeletal and dental phenotype in the *Alpl* ^*Prx1/Prx1*^ and *Phospho1* ^−/−^ mouse models of late‐onset (adult) HPP and pseudo‐HPP, respectively. A single dose of 3 × 10^11^ vector genomes per body (vg/b) was injected intramuscularly into 8‐week‐old *Alpl* ^*Prx1/Prx1*^ and wild‐type (WT) littermates, or into 3‐day‐old *Phospho1* ^−/−^ and WT mice, and treatment efficacy was evaluated after 60 days for late‐onset HPP mice and after 90 days for *Phospho1* ^−/−^ mice. Biochemical analysis showed sustained serum alkaline phosphatase activity and reduced plasma PP_i_ levels, and radiographic images, micro‐computed tomography (micro‐CT) analysis, and hematoxylin and eosin (H&E) staining showed improvements in the long bones in the late‐onset HPP mice and corrected scoliosis in the *Phospho1* ^*−/−*^ mice. Micro‐CT analysis of the dentoalveolar complex did not reveal significant changes in the phenotype of late‐onset HPP and pseudo‐HPP models. Moreover, alizarin red staining analysis showed that AAV8‐TNAP‐D_10_ treatment did not promote ectopic calcification of soft organs in adult HPP mice after 60 days of treatment, even after inducing chronic kidney disease. Overall, the AAV8‐TNAP‐D_10_ treatment improved the skeletal phenotype in both the adult HPP and pseudo‐HPP mouse models. This preclinical study will contribute to the advancement of gene therapy for the improvement of skeletal disease in patients with heritable forms of osteomalacia. © 2022 The Authors. *JBMR Plus* published by Wiley Periodicals LLC on behalf of American Society for Bone and Mineral Research.

## Introduction

Hypophosphatasia (HPP), a rare genetic metabolic disorder, mainly affects skeletal and dental mineralization due to loss‐of‐function mutation(s) in the *ALPL* gene that encodes tissue‐nonspecific alkaline phosphatase (TNAP).^(^ ^1^, ^2^ ^)^ The adult form of HPP usually presents during middle age. Although most patients may not recall childhood symptoms, there are reports of childhood delay in crawling, difficulty walking or climbing stairs, unusual or waddling gait, muscle weakness, reduced activity in childhood, poor muscle tone, bone and muscle pain, seizures, and poor dentition. Patients may manifest recurrent fractures, including delayed healing, that start occurring in adult life.^(^ ^3^, ^4^ ^)^ Adult HPP patients may present with nonspecific musculoskeletal manifestations, such as bone and joint pain as a frequent complaint from ectopic calcification in the joints, joint inflammation, as well as slow‐healing pseudo fractures or fractures with slow healing that leads to pseudoarthrosis.^(^ ^5^ ^)^ Other manifestations of HPP in adults include osteomalacia,^(^ ^2^, ^3^ ^)^ osteopenia,^(^ ^2^ ^)^ short stature,^(^ ^6^ ^)^ muscle weakness, unusual gait, kidney stones,^(^ ^7^ ^)^ impaired mobility,^(^ ^3^ ^)^ adult tooth loss,^(^ ^3^, ^8^ ^)^ abnormal dentition, and periodontal disease.^(^ ^5^ ^)^ Those several manifestations are often misdiagnosed as other disorders.

Phosphoethanolamine/phosphocholine phosphatase (PHOSPHO1), present in the lumen of matrix vesicles, plays a critical role in initiating bone matrix mineralization. Studies in PHOSPHO1‐deficient mice (*Phospho1* ^−/−^) display elevated PP_i_ levels and decreased plasma TNAP activity^(^ ^9^, ^10^, ^11^ ^)^ and present skeletal abnormalities, including fractures, bowed long bones, osteomalacia, and scoliosis in the early stage of life that resemble HPP, and thus are here referred to as a model of pseudo‐HPP.^(^ ^11^, ^12^, ^13^ ^)^ The pathophysiology of rickets/osteomalacia and dental disease in HPP and *Phospho1* ^−/−^ mice can be explained by the accumulation in the extracellular matrix of PP_i_, a potent mineralization inhibitor^(^ ^14^ ^)^ and one of the natural substrates of TNAP.^(^ ^15^, ^16^, ^17^ ^)^

Asfotase alfa, a recombinant form of TNAP (sALP‐Fc‐D_10_) that contains the Fc region of immunoglobulin (Fc) and a deca‐aspartate (D_10_) mineral‐targeting motif^(^ ^18^ ^)^ was shown efficacious in extending life and preventing skeletal and dental disease in a mouse model of severe infantile HPP.^(^ ^18^, ^19^, ^20^, ^21^, ^22^ ^)^ These studies led to clinical trials in infants and young children with perinatal/infantile HPP,^(^ ^23^ ^)^ and this biologic was subsequently approved in 2015 as an enzyme replacement therapy (ERT) for pediatric‐onset HPP in the US and Europe and regardless of age of onset in Japan. ERT with asfotase alfa requires subcutaneous injections from 3 to 6 times a week, which are expected to continue for life.^(^ ^23^ ^)^

Gene therapy is a powerful approach to the treatment of several genetic diseases. Recent studies have shown that a single administration of either a lentiviral vector or adeno‐associated viral vectors expressing TNAP‐D_10_ caused continued expression of TNAP and the improvement in bone and dentoalveolar phenotype in a severe infantile HPP mouse model.^(^ ^24^, ^25^, ^26^, ^27^, ^28^, ^29^, ^30^ ^)^ This gene therapy method is referred to as viral vector‐mediated ERT and is administered as a single injection, unlike traditional ERT, which requires recurrent injections. However, this approach has not been studied for treating adult forms of HPP. Here, we evaluated the efficacy of viral‐mediated ERT using adeno‐associated virus vector serotype 8 (AAV8) harboring mineral‐targeted TNAP (AAV8‐TNAP‐D_10_) for a mouse model of adult HPP and as well as the *Phospho1*‐deficient mouse model of pseudo‐HPP.

## Materials and Methods

### Mouse models of late‐onset HPP and pseudo‐HPP

*Prx1‐Cre* ^*+/−*^; *Alpl* ^*flox/flox*^ mice (hereon called *Alpl* ^*Prx1/Prx1*^), a murine model of adult HPP, was generated as previously described via the conditional ablation of *Alpl* in mesenchymal cells through the expression of Cre recombinase under the control of the *Prx1* promoter.^(^ ^31^ ^)^ Wild‐type (WT) *Alpl* ^*flox/flox*^ littermates were used as controls. We crossed *Alpl* ^*flox/flox*^ mice to *Prx1 Cre* ^*+/−*^ heterozygous mice to generate *Prx1‐Cre* ^*+/−*^; *Alpl* ^*flox/+*^ animals and then they were bred to produce *Prx1‐Cre* ^*+/−*^; *Alpl* ^*flox/flox*^ mice. *Prx1‐Cre*; *Alpl* ^*flox/flox*^ line has a hybrid background of C57BL/6 and SJL/J. The conditional Prx1‐Cre; *Alpl* ^*flox/flox*^ line is a bone‐specific knockout since the Prx‐Cre gene is expressed in chondrocytes, osteoblasts, and mesenchymal stem cells. The adult HPP model shows a milder phenotype in bones than constitutive Alpl^−/−^ mice, and there is no observation of pain or distress. Prx1‐Cre; *Alpl* ^*flox/flox*^ mice are viable and apparently live a normal life span.

Phospho1‐R74X null mutant (*Phospho1* ^−/−^, *Phospho1* KO) mouse, a model of pseudo‐HPP, was generated by *N*‐ethyl‐*N*‐nitrosourea mutagenesis (ENU) in the C3HeB/FeJ background (The Jackson Laboratory, Bar Harbor, ME, USA) and bred to C57BL/6 mice as previously reported.^(^ ^19^ ^)^ The *Phospho1* KO animals live a normal life span. Despite the fact that *Phospho1* ^*−/−*^ mice develop skeletal abnormalities, they do not appear to suffer from pain and they are fertile.

### Animal care

This animal study was approved by the Institutional Animal Care and Use Committee at Sanford Burnham Prebys Medical Discovery Institute (#20–090; #19–065). The mice were bred and maintained according to animal facility breeding standard operating procedures (SOPs). Breeding of animals was done by vivarium personnel according to standard vivarium policies. The animal room had a 12‐hour light/dark cycle, and it was kept clean and pathogen‐free. Mice were housed in groups of 5 maximum. Animals were separated if there was evidence of fighting. Certified Envigo Teklad (Indianapolis, IN, USA) Global Rodent Diets were available *ad libitum* during acclimation and throughout the study. The 2019 Teklad Global Diet diet was used for breeding and the Envigo Teklad Global Diet 2018 was used for holding mice. For chronic kidney disease (CKD) experiments, Teklad Custom Diet 0.2% Adenine (2018) (TD.09138) and Teklad Custom Diet 1.8% Total P, 0.2% Adenine (2018) (TD.190831) were used. Sterile deionized water was available *ad libitum* from individual bottles attached to the cages. Each animal was given a sequential number by ear notches or toe clipping. Each cage was identified by a cage card indicating at least the study number and starting date, individual animal identifications, date of birth (DOB), sex of the animals, and group assignment.

This study complied with the applicable sections of the Final Rules of the Animal Welfare Act Regulations and the most recent Guide for the Care and Use of Laboratory Animals. Procedures used in this study were designed to avoid or minimize discomfort, distress, or pain to animals. Animals that experienced severe or chronic pain or distress that could not be relieved were painlessly euthanized as deemed appropriate by the animal facility staff or the study performer. The methods of euthanasia used during this study, ie, exsanguination with anesthesia or CO_2_, were conformed to the regulations cited above and the American Veterinary Medical Association (AVMA) Guidelines on Euthanasia. When appropriate, anesthesia was done intraperitoneally with Avertin (0.017 μL/g body weight).

### Virus vector encoding the human TNAP‐D_10_ cDNA

Recombinant AAV8 encoding mineral‐targeted TNAP (AAV8‐TNAP‐D_10_; aka ARU‐2801) was generated using the HEK293 cell line as previously reported.^(^ ^32^, ^33^, ^34^ ^)^ As a control, we used a recombinant AAV8 vector encoding the green fluorescent protein (GFP) (AAV8‐GFP).^(^ ^35^ ^)^ Eight‐week‐old *Alpl* ^*Prx1/Prx1*^ and WT sibling mice, as well as *Phospho1* ^−/−^ and WT littermates within 3 days of age, received a single intramuscular injection of AAV8‐TNAP‐D_10_ or AAV8‐GFP vector as control at a dose of 3 × 10^11^ vector genomes (vg)/body into the quadriceps femoris as previously reported.^(^ ^28^ ^)^

### Induction of uremia

To assess whether AAV8‐TNAP‐D_10_ worsens ectopic calcification, we induced chronic kidney disease in mice. Eight‐week‐old females *Alpl* ^*Prx1/Prx1*^ and WT siblings mice were fed with a diet containing adenine (0.2%) for 4 weeks, and adenine + high phosphorus diet (0.2% adenine and 1.8% phosphorus) for another 4 weeks,^(^ ^36^ ^)^ to induce a state of uremia that recapitulates CKD, which leads to vascular calcification. The animals were monitored weekly by measuring body weight and observing clinical signs such as posture and gait, difficulty eating, etc. Animals received a single dose of AAV8‐TNAP‐D_10_ or AAV8‐GFP treatment on the same day as the induction of uremia. After 8 weeks, mice were euthanized, and soft organs, including kidneys, aorta, and heart, were harvested from each animal to assess the status of calcification. Mice were daily monitored on modified diets and promptly euthanized when they showed signs of distress (poor dietary intake, lack of self‐care, lack of movement).

### Biochemical analysis

Body weight of the mice was first measured and then they were anesthetized with isoflurane for blood (plasma/serum) collection via orbital sinus of mice using Pasteur pipets before virus vector treatment and 60 days after injection for late‐onset HPP mice, and after 45 and 90 days of treatment for the pseudo‐HPP mice. Blood samples were centrifuged at 5200 *g* for 10 minutes. For PP_i_ analysis, a deproteinization method was first performed by adding 20 μL of plasma to a Microcon‐10 kDa Centrifugal Filter Unit with Ultracel‐10 membrane (MilliporeSigma, Merck KGaA, Darmstadt, Germany), and then centrifuged at 14,000*g* for 20 minutes.

Plasma PP_i_ concentrations were measured according to the protocol reported previously.^(^ ^36^ ^)^ Deproteinized plasma sample (5 uL) was added to 45 μL of assay mixture containing 90 μM adenosine 5′ phosphosulfate sodium salt (APS) (Sigma‐Aldrich, St. Louis, MO, USA), 22.5 μM MgCl_2_, 11.25 mM HEPES with 0.9 U/mL recombinant Yeast ATP‐sulfurylase/MET3 (R&D Systems, Inc., Minneapolis, MN, USA). PP_i_ standards ranged from 20 to 0.125 μM (sodium pyrophosphate decahydrate, Sigma‐Aldrich). The combination was incubated at 37°C for 30 minutes and heat‐inactivated at 90°C for 10 minutes. Then 10 μL of each sample was transferred into a 96‐well plate and mixed with 50 μL of BacTiter‐Glo Microbial Cell Viability Assay (Promega Corporation, Madison, WI, USA). Endpoint, luminescence was measured at 578 nm by the microplate reader FilterMax F5 Multimode (VWR International, LLC, Radnor, PA, USA). Serum ALP activity was performed by adding 5 μL of serum and recombinant human ALP standards (216.0–0.099 ng/mL) into a 96‐well plate, and then 95 μL of 10 mM pNPP in diethanolamine (DEA) buffer (pH 9.8) containing 1.0 mM MgCl_2_ and 20 μM ZnCl_2_ was added. The absorbance of the kinetics assay was measured at 405 nm using OptiMax Microplate Absorbance Reader (Molecular Devices, LLC, San Jose, CA, USA) for 30 minutes. To measure both serum calcium and blood urea nitrogen (BUN) levels, QuantiChrom Calcium Assay Kit and QuantiChrom Urea Assay Kit (BioAssay Systems, Hayward, CA, USA) were used, respectively. To measure serum phosphorus concentrations, Stanbio Phosphorus Liqui‐UV (EKF Diagnostics‐Stanbio Laboratory, Boerne, TX, USA) was used according to the manufacturer.

### Radiography and micro‐computed tomography (micro‐CT)

Radiographic images of the whole skeleton as well as the isolated bones, including the cranium, vertebrae, long bones, and hemimandibles, were acquired using the energy of 20 kV, with the Faxitron MX‐20DC4 radiograph system (Chicago, IL, USA).

Before micro‐CT analysis, long bones and right hemimandibles were fixed in 4% paraformaldehyde/PBS solution. Femurs were scanned using a SkyScan 1172 scanner (Bruker Micro‐CT, Kontich, Belgium) at 55 kV, 181 μA, 0.5 mm Al filter, 280 ms integration time, and 10 μm voxel dimension. Micro‐CT images were reconstructed in NRecon software and were calibrated to three known densities of hydroxyapatite. Analysis was performed using AnalyzePro (version 1.0; AnalyzeDirect, Overland Park, KS, USA). Trabecular and cortical bone of femurs were segmented at 400 and 550 mg hydroxyapatite (HA)/cm^3^, respectively. Micro‐CT analysis of femurs was performed as previously described.^(^ ^28^ ^)^ Briefly, to quantify the trabecular bone parameters, such as bone volume (Tb.BV), total volume (Tb.TV), bone volume fraction (Tb.BV/TV), trabecular number (Tb.N), thickness (Tb.Th), spacing (Tb.Sp), connective density (Tb.Conn.D), and mineral density (Tb.BMD), a total of 0.5 mm (50 slices) proximal to the distal femur growth plate was traced. For the cortical bone, 50 slices of the mid‐femur of each bone were used to quantify cortical bone volume fraction (Ct.BV/TV), cortical thickness (Ct.Th), marrow area (Ma.Ar), and mineral density (Ct.BMD).^(^ ^28^ ^)^

Hemimandibles were scanned using a μCT 50 scanner (Scanco Medical, Bassersdorf, Switzerland) at 70 kV, 76 μA, 0.5 mm Al filter, 900 ms integration time, and 6 μm voxel dimension. Reconstructed images were calibrated to five known densities of HA and analyzed using AnalyzePro Software (version 1.0; AnalyzeDirect). Micro‐CT analysis of the first mandibular molar and associated alveolar bone was performed as previously described.^(^ ^37^ ^)^ The region of interest of alveolar bone was defined to include 240 μm mesial to the most mesial point of the first molar mesial root and 240 μm distal to the most distal point of the distal root. Alveolar bone and dentin/cementum were segmented at 650 to 1600 mg/cm^3^ HA, whereas enamel was segmented above 1600 mg/cm^3^ HA.

### Tissue collection and processing for histological studies

Treated *Alpl* ^*Prx1/Prx1*^ and *Phospho1* ^*−/−*^ mice were anesthetized with an intraperitoneal administration of Avertin and euthanized by exsanguination after 60 and 90 days, respectively. Skeletal/dental tissues were collected, fixed in 4% paraformaldehyde/PBS or Bouin's solution, and processed for histological analysis. The long bones and vertebrae were placed in 0.125 M EDTA/10% formalin (pH 7.3) solution for 7 days for decalcification and then were paraffin‐embedded and sectioned at 5 μm thickness. Hemimandibles were fixed in Bouin's solution, decalcified in an acetic acid/formalin/sodium chloride solution, processed for paraffin embedding, and sectioned at 6 μm thickness. Hematoxylin and eosin (H&E) and alizarin red staining were performed according to standard methods. Aperio AT2 system (Leica Biosystems of Leica Microsystems Inc., Buffalo Grove, IL, USA) was used to scan the slides. The quantification of alizarin red staining was done by Aperio Color Deconvolution Algorithm Software (Leica Biosystems). Calcified sites are shown as a percentage of strong positive alizarin red staining.

### Statistical analysis

Statistical analyses were performed using one‐way ANOVA followed by Tukey's multiple comparisons test for comparisons with more than two groups or unpaired *t* test for comparisons between two groups by GraphPad Prism version 9.0.0 (GraphPad Software, San Diego, CA, USA). Values were expressed as the mean ± SD. Differences were statistically significant at *p < 0.05.

## Results

### Biochemical analysis of mice under AAV8‐TNAP‐D_10_ treatment

**Fig. 1:**
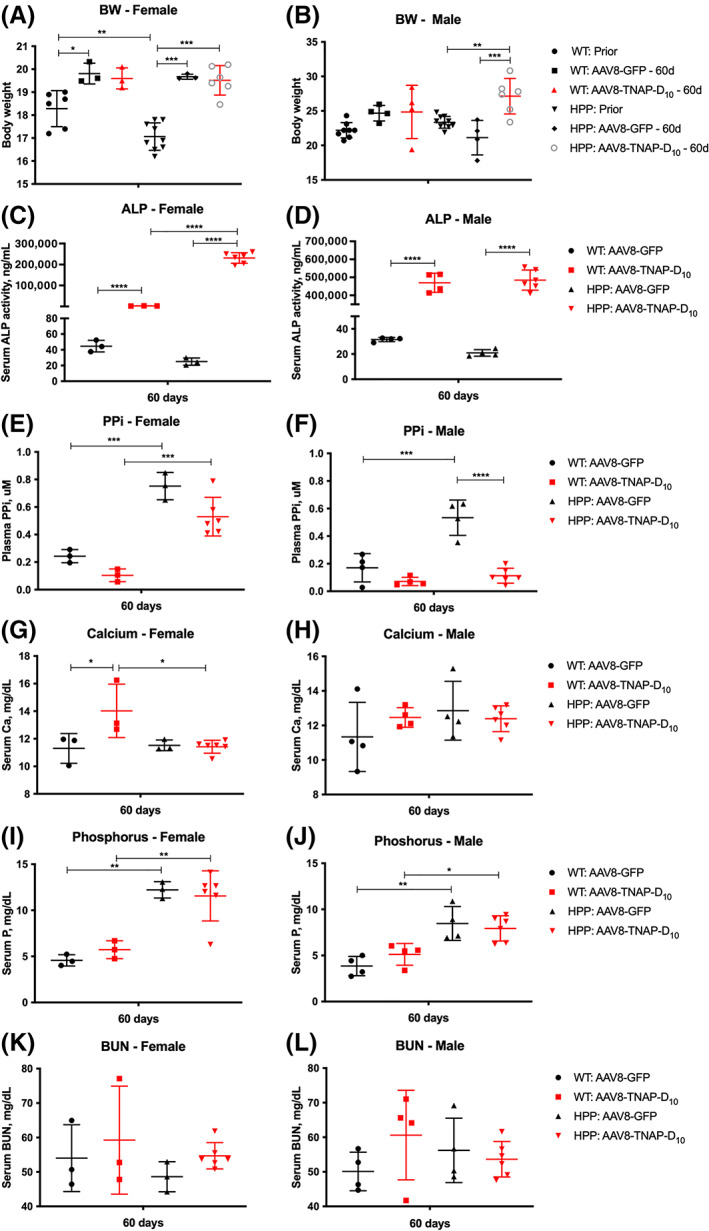
Biochemical analysis in serum/plasma from adult hypophosphatasia (HPP) mice and wild‐type (WT) littermates under AAV8‐TNAP‐D_10_ treatment or AAV8‐GFP control, 60 days after injection. (*A*, *B*) Body weight. (*C*, *D*) Serum alkaline phosphatase activity. (*E*, *F*) Plasma PP_i_ levels. (*G*, *H*) Serum calcium concentrations. (*I*, *J*) Serum phosphorus concentrations. (*K*, *L*) Blood urea nitrogen (BUN) levels in serum. Sample size of the cohorts was as follows: (i) HPP: AAV8‐TNAP‐D_10_ female *n* = 6, male *n* = 6; (ii) HPP: AAV8‐GFP female *n* = 3, male *n* = 4; (iii) WT: AAV8‐TNAP‐D_10_ female *n* = 3, male *n* = 4; (iv) WT: AAV8‐GFP female *n* = 3, male *n* = 4. Statistical analysis was performed by one‐way ANOVA followed by Tukey's multiple comparison test. *p < 0.05; **p < 0.01; ***p < 0.001; ****p < 0.0001.

We monitored body weight and biochemical markers in serum and/or plasma from adult HPP and WT mice before treatment (at 8 weeks of age) and 60 days after the AAV8‐TNAP‐D_10_ injection. Body weights of female adult HPP mice were slightly lower than WT littermates before the injection. After 60 days of treatment, there was an increase in body weight, whereas in males before treatment, no differences were observed. Male adult HPP mice showed body weight increase after AAV8‐TNAP‐D_10_ treatment when compared with HPP AAV8‐GFP (vehicle)‐injected mice (Fig. 1*A*, *B*). Serum alkaline phosphatase (ALP) activity results were significantly lower and PP_i_ was significantly higher in female and male adult HPP mice than WT mice before treatment (Supplemental Fig. S1 *A–D*; Supplemental Table S1). There were no differences in serum calcium, BUN, or phosphorus levels in HPP and WT mice before treatment, except for female HPP, in which we observed lower phosphorus levels when compared with WT mice before injection (Supplemental Fig. S1 *E–J*; Supplemental Table S1).

At 60 days after administering AAV8‐TNAP‐D_10_, serum ALP activity was significantly higher in female and male adult HPP mice and in WT mice compared with the vehicle group (Fig. 1*C*, *D*; Supplemental Table S2). Plasma PP_i_ concentrations were higher in both female and male adult HPP mice when compared with WT in the control AAV8‐GFP‐treated mice. Plasma PP_i_ levels were significantly lower in male adult HPP AAV8‐TNAP‐D_10_‐treated mice but not in female AAV8‐TNAP‐D_10_‐treated mice compared with vehicle‐treated adult HPP mice (Fig. 1 *E*, *F*; Supplemental Table S2). AAV8‐TNAP‐D_10_ treatment increased the calcium levels in WT females but not males. The calcium levels of adult HPP mice were not affected by the TNAP treatment (Fig. 1*G*, *H*; Supplemental Table S2). High phosphorus levels were observed in adult HPP mice treated with vehicle or with AAV8‐TNAP‐D_10_ (Fig. 1*I*, *J*; Supplemental Table S2). There were no significant differences in BUN from serum samples of HPP and WT mice treated with AAV8‐TNAP‐D_10_ and AAV8‐GFP (Fig. 1 *K*, *L*; Supplemental Table S2).

**Fig. 2:**
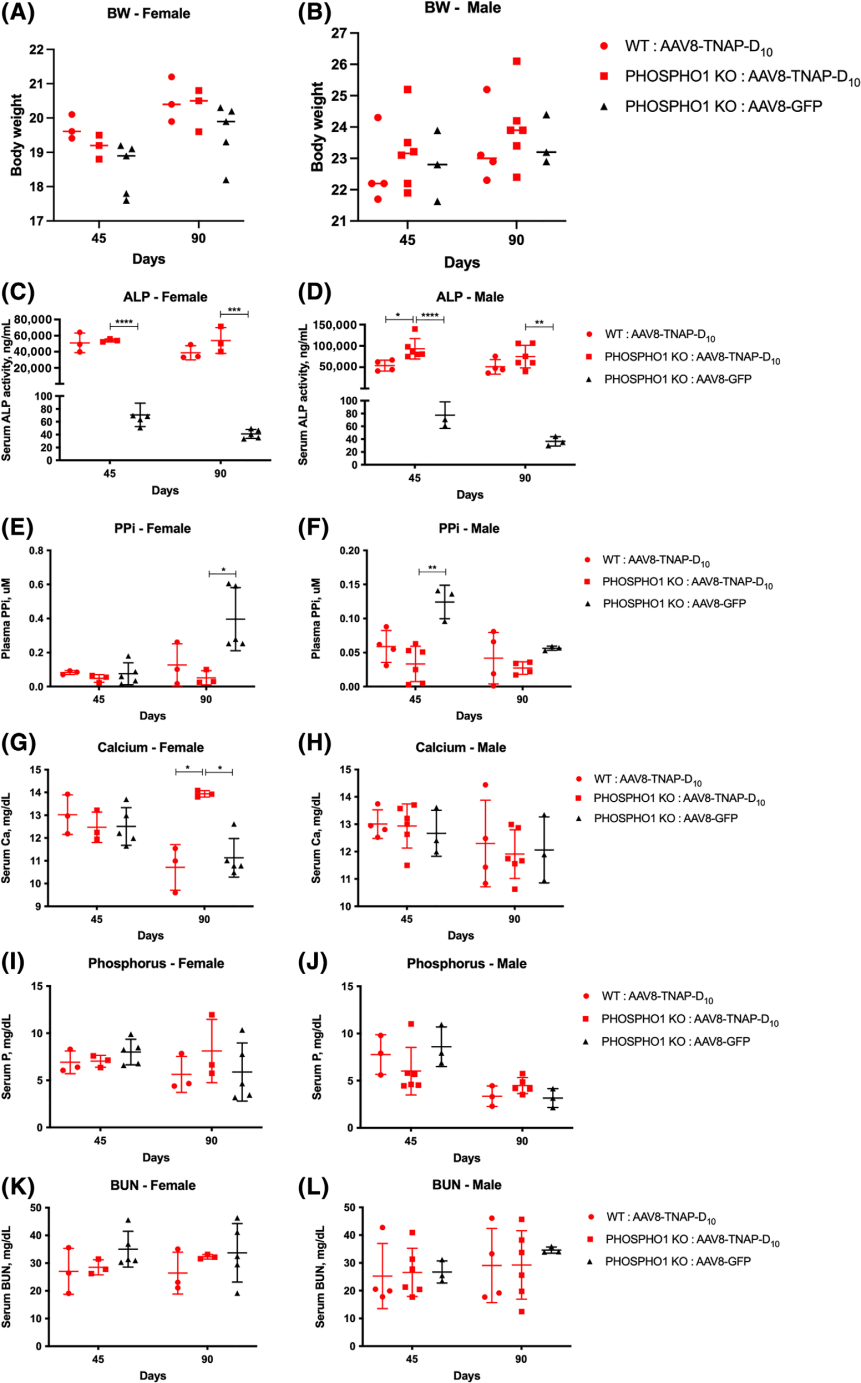
Biochemical analysis in serum/plasma from *Phospho1* knockout (KO) mice under AAV8‐TNAP‐D_10_ treatment or AAV8‐GFP as control after 45 and 90 days of injection. Wild‐type (WT) littermates were treated with the AAV8‐TNAP‐D_10_ vector. (*A*, *B*) Body weight. (*C*, *D*) Serum alkaline phosphatase activity. (*E*, *F*) Plasma PP_i_ levels. (*G*, *H*) Serum calcium concentrations. (*I*, *J*) Serum phosphorus concentrations. (*K*, *L*) Blood urea nitrogen (BUN) levels in serum. Sample size of the cohorts was as follows: (i) *Phospho1* KO: AAV8‐TNAP‐D_10_ female *n* = 3, male *n* = 6; (ii) *Phospho1* KO: AAV8‐GFP female *n* = 5, male *n* = 3; (iii) WT: AAV8‐TNAP‐D_10_ female *n* = 3, male *n* = 4. Statistical analysis was performed by one‐way ANOVA followed by Tukey's multiple comparison test. *p < 0.05; **p < 0.01; ***p < 0.001. ****p < 0.0001.

Body weight from *Phospho1* ^*−/−*^ and WT mice were measured 45 and 90 days after the AAV8‐TNAP‐D_10_ injection. Body weights from females and males did not show significant statistical differences (Fig. 2 *A*, *B*). Biochemical markers were assessed in *Phospho1* ^−/−^ mice 45 and 90 days after AAV8‐TNAP‐D_10_ treatment. Serum ALP activity was significantly higher and plasma PP_i_ levels were lower in both female and male AAV8‐TNAP‐D_10_‐treated mice compared with AAV8‐GFP‐treated mice (Fig. 2*C–F*; Supplemental Table S3). The levels of calcium in female but not male *Phospho1* ^−/−^ mice were increased after 90 days of AAV8‐TNAP‐D_10_ injection when compared with the control AAV8‐GFP treatment and AAV8‐TNAP‐D_10_‐treated WT mice (Fig. 2*G*, *H*; Supplemental Table S3). No significant differences in phosphorus or BUN levels were observed after AAV8‐TNAP‐D_10_ treatment (Fig. 2*I–L*; Supplemental Table S3).

### Amelioration of the skeletal abnormalities with AAV8‐TNAP‐D_10_ treatment without evidence of ectopic calcification in soft organs

**Fig. 3:**
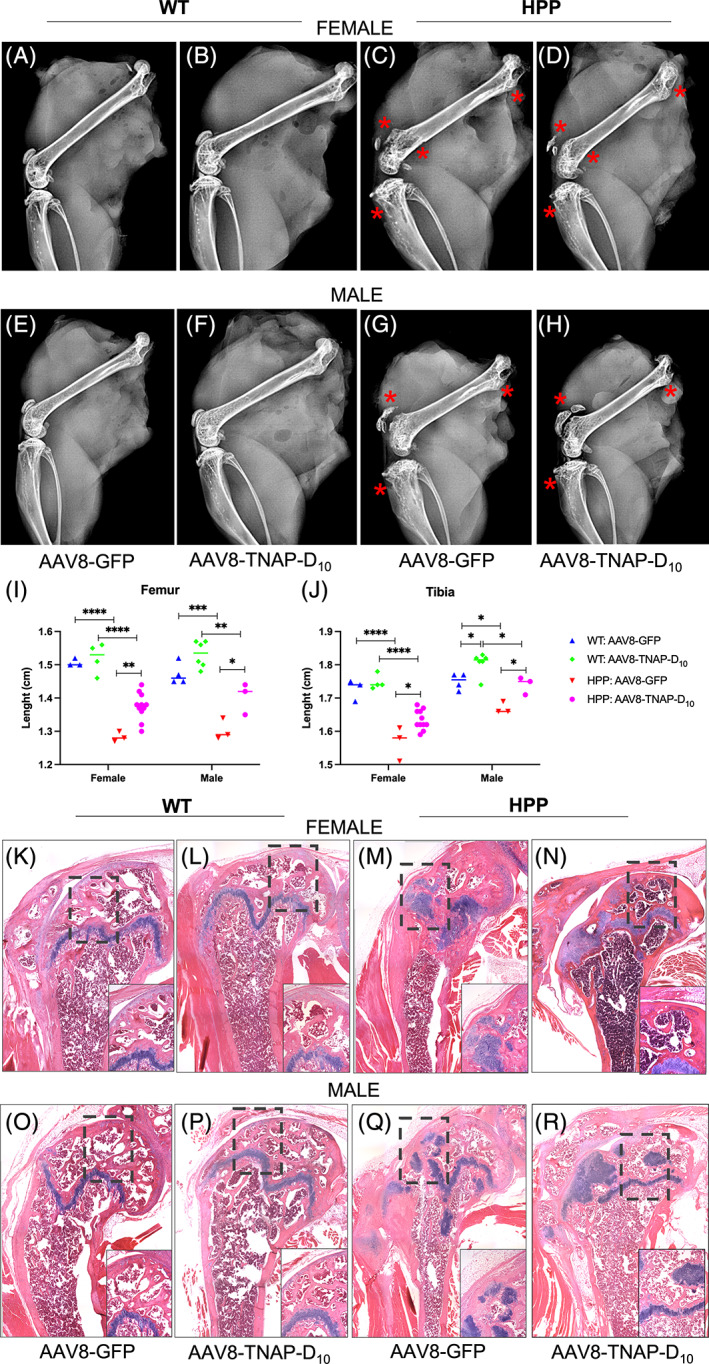
Radiographic findings and hematoxylin and eosin (H&E) staining of long bones in adult hypophosphatasia (HPP) mice bone phenotype. X‐ray images from (*A*–*D*) female and (*E*–*H*) male HPP mice and wild‐type (WT) littermate control treated with AAV8‐TNAP‐D_10_ or AAV8‐GFP after 60 days of injection. Impaired long bones from (*C*, *G*) HPP mice with defects in the proximal and distal femur along with the patellar joint surface and proximal tibia. (*D*–*H*) TNAP treatment partially rescued the epiphyseal and metaphyseal bone regions (highlighted in red). (*I*) Femur and (*J*) tibia length measurement. (K‐O) H&E staining showing the growth plate in AAV8‐GFP and (L‐P) in AAV8‐TNAP‐D_10_ ‐treated WT mice. (*M*–*Q*) H&E staining showed the disorganization of the growth plate in AAV8‐GFP‐treated HPP mice and (*N*–*R*) a substantial improvement of the bone morphologic parameters in AAV8‐TNAP‐D_10_‐treated HPP mice. H&E images were acquired in tiling mode and 20× magnification. Statistical analysis was performed by one‐way ANOVA followed by Tukey's multiple comparison test. *p < 0.05. **p < 0.01. ***p < 0.001. ****p < 0.0001.

Radiographic images of female and male adult HPP mice revealed normal skeletal development when compared to WT littermates (Supplemental Figs. S2 *A–D* and S3 *A–D*). However, long bones of female and male adult HPP mice presented defects in the epiphyseal and metaphyseal regions of the femur and tibia along with the patellar joint surface when compared with WT mice (Fig. 3*A*, *C*, *E*, *G*). Long bones of AAV8‐TNAP‐D_10_‐treated adult HPP mice were partially rescued as observed by the improvement of the proximal tibia and distal femur regions as well as by the femur and tibia lengths when compared with the AAV8‐GFP‐treated adult HPP mice (Fig. 3*C*, *D*, *G–J*). In addition, there were no changes in the bone phenotype in AAV8‐TNAP‐D10‐treated WT mice when compared with AAV8‐GFP‐treated WT mice (Fig. 3*A*, *B*, *E*, *F*). Histological sections of femurs stained with H&E from female and male adult HPP mice confirmed the impaired area visualized through X‐ray images when compared with WT mice. A disorganized growth plate with a modified chondrocyte columnar organization was observed in adult HPP mice but not in WT mice (Fig. 3*K*, *M*, *O*, *Q*). Femurs of AAV8‐TNAP‐D_10_‐treated adult HPP mice presented preserved growth plate area as indicated by the bone morphological organization related to the AAV8‐GFP‐treated HPP mice (Fig. 3*M*, *N*, *Q*, *R*). In addition, no histological changes in the bone phenotype in AAV8‐TNAP‐D_10_‐treated WT mice were observed when compared with WT mice treated with AAV8‐GFP (Fig. 3*K*, *L*, *O*, *P*).

**Fig. 4:**
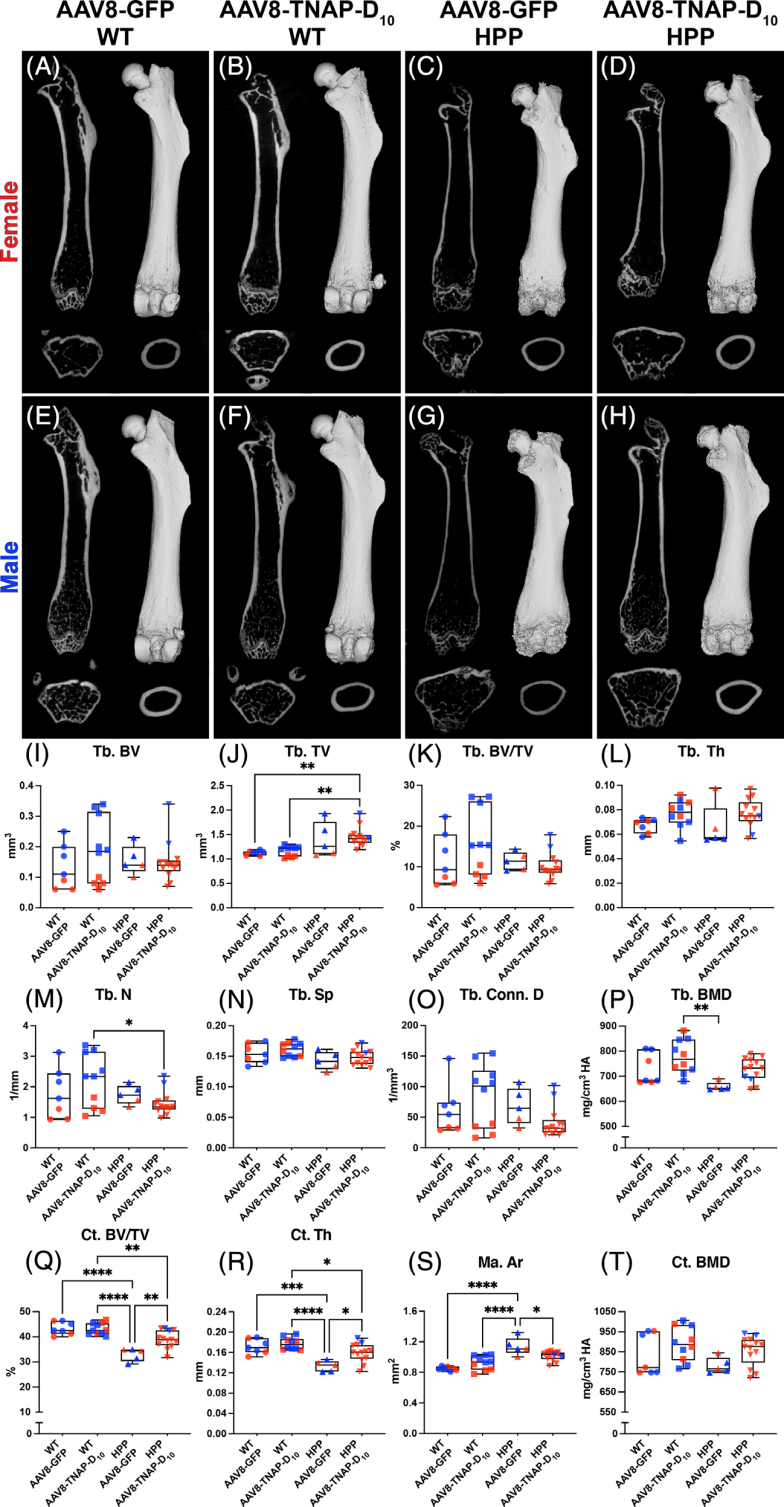
Micro‐CT analysis of femur from adult hypophosphatasia (HPP) mice and wild‐type (WT) littermates. 2D and 3D micro‐CT images of femurs from (*A*–*D*) females and (*E*–*H*) males treated with AAV8‐TNAP‐D_10_ or AAV8‐GFP after 60 days of injection. (*I*–*T*) Micro‐CT analysis of bone parameters in the femurs showing reduced cortical bone volume fraction (Ct.BV/TV) associated with a reduced cortical thickness (Ct.Th) and increased marrow area (Ma.Ar) in untreated HPP mice compared with AAV8‐TNAP‐D_10_ or AAV8‐GFP WT controls. These cortical bone defects were partially rescued after 60 days of AAV8‐TNAP‐D_10_ treatment. Statistical analysis was performed by one‐way ANOVA followed by Tukey's multiple comparison test. Differences were statistically significant at *p < 0.05. **p < 0.01. ***p < 0.001. ****p < 0.0001.

Micro‐CT analysis of bone parameters showed that untreated HPP mice had a statistically significant reduction in cortical bone volume fraction (Ct.BV/TV) compared with untreated and AAV8‐TNAP‐D_10_‐treated WT littermates. The reduced cortical BV/TV in untreated HPP mice was associated with a significantly reduced cortical thickness (Ct.Th) and increased marrow area (Ma.Ar) versus untreated and treated WT littermates. However, the cortical bone mineral density (Ct.BMD) of untreated HPP mice was not statistically significant different from treated and untreated WT littermates. Gene therapy using the AAV8‐TNAP‐D_10_ vector significantly improved cortical bone in HPP mice, including bone fraction (Ct.BV/TV), thickness, and marrow area. Notably, no statistically significant difference was observed between treated and untreated WT mice, suggesting no adverse effects of AAV8‐TNAP‐D_10_ vector‐mediated gene therapy over 60 days (Fig. 4*A–T*).

**Fig. 5:**
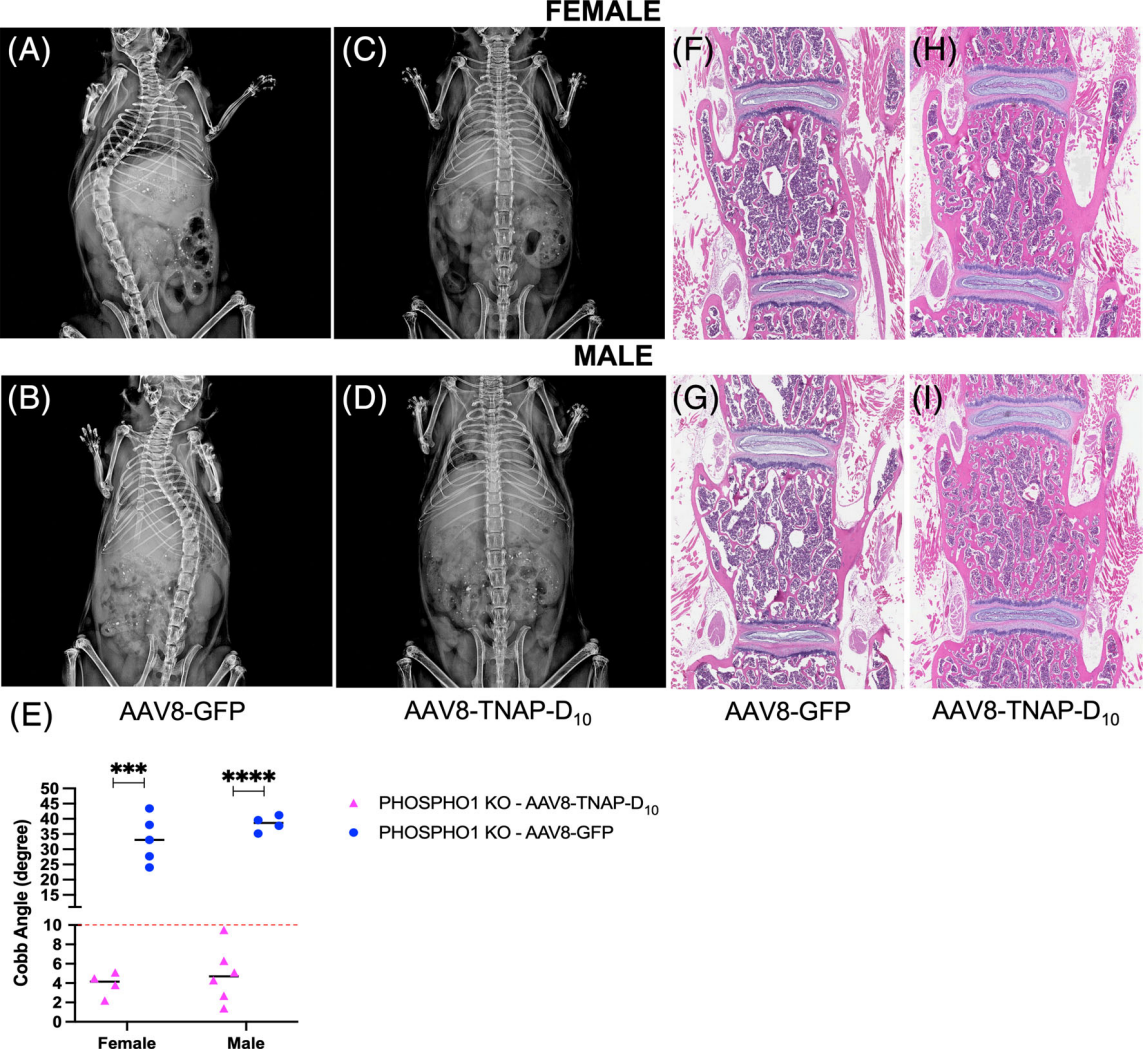
Representative X‐ray images and histological sections of the spine from female and male 90‐day‐old *Phospho1* knockout (KO) mice. A single dose of AAV8‐TNAP‐D_10_ or AAV8‐GFP, as control (3 × 10^11^ vg/body), was intramuscularly administered in 3‐day‐old mice. (*A*, *B*) The control AAV8‐GFP‐treated *Phospho1* KO mice showed scoliosis. (*C*, *D*) Gene therapy using the AAV8‐TNAP‐D_10_ vector corrected the scoliosis deformity of the spine. (*E*) Cobb angle measurements. The threshold of scoliosis (Cobb angle >10°) was indicated with a dashed red line. Hematoxylin and eosin (H&E) staining showed a different tissue organization of vertebrae from mice treated with (*F*, *G*) AAV8‐GFP or (*H*, *I*) the vector encoding TNAP, revealing increased trabecular bone and less bone marrow area in AAV8‐TNAP‐D_10_‐treated mice. H&E images were acquired in 10× magnification.

**Fig. 6:**
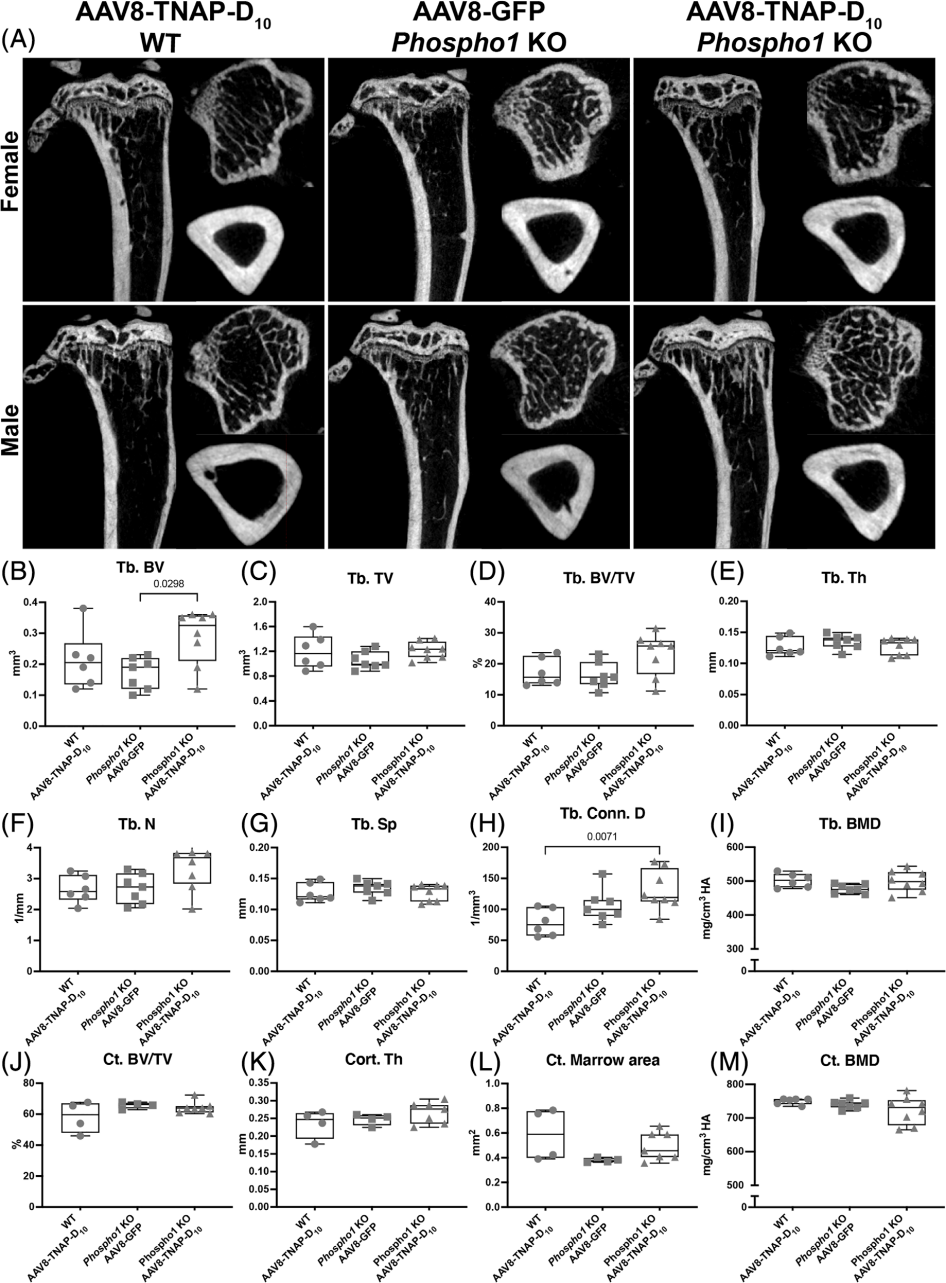
Micro‐CT analysis of tibias from *Phospho1* knockout (KO) mice and wild‐type (WT) littermates. (*A*) 2D micro‐CT images of tibias from females and males treated with AAV8‐TNAP‐D_10_ or AAV8‐GFP 90 days after injection. (*B*–*I*) Quantification of trabecular bone parameters showing increased bone volume and trabecular bone connectivity in AAV8‐TNAP‐D_10_‐treated *Phospho1* KO compared with WT and untreated *Phospho1* KO mice. (*J*–*M*) quantification of cortical bone parameters showed no statistically significant difference among the groups. Statistical analysis was performed by one‐way ANOVA followed by Tukey's multiple comparison test. Differences were statistically significant at *p < 0.05 (*p* = 0.0298) and **p < 0.01 (*p* = 0.0071).

Radiographic images of female and male *Phospho1* ^−/−^ mice showed apparent normal skeletal development, except for a spinal deformity, ie, the abnormal moderate and severe degrees of scoliosis in vertebrae of untreated *Phospho1* ^−/−^ mice (Supplemental Figs. S4 *A*, *B*, and S5 *A*, *B*). Interestingly, a single dose of AAV8‐TNAP‐D_10_ improved the scoliotic curves and corrected this congenital manifestation in female and male *Phospho1* ^−/−^ mice as found 90 days after injection, observed by X‐rays images and the Cobb angle measurements (Fig. 5*A–E*). H&E staining of the fourth lumbar vertebrae showed more trabecular bone and a reduced marrow area in AAV8‐TNAP‐D_10_‐treated mice when compared with AAV8‐GFP control (Fig. 5*F–I*). Micro‐CT analysis of tibias in female and male AAV8‐TNAP‐D_10_ *Phospho1* ^−/−^‐treated mice showed increased trabecular bone volume (Tb.BV) and trabecular connectivity density (Tb.Conn.D) when compared with AAV8‐GFP *Phospho1* ^−/−^‐treated mice and AAV8‐TNAP‐D_10_ WT mice, respectively (Fig. 6).

In addition, the treatment with the AAV8‐TNAP‐D_10_ vector did not promote ectopic calcification in the kidney, liver, heart, or aorta of female or male adult HPP mice (Supplemental Fig. S6 *A*, *B*) in this short treatment window (60 days of treatment), and it did not increase the calcification of soft organs in adult HPP model after 60 days of treatment when chronic kidney disease was superimposed as a comorbidity (Supplemental Fig. S7 *A–M*).

### Characterization of dentoalveolar complex in both osteomalacia models

**Fig. 7:**
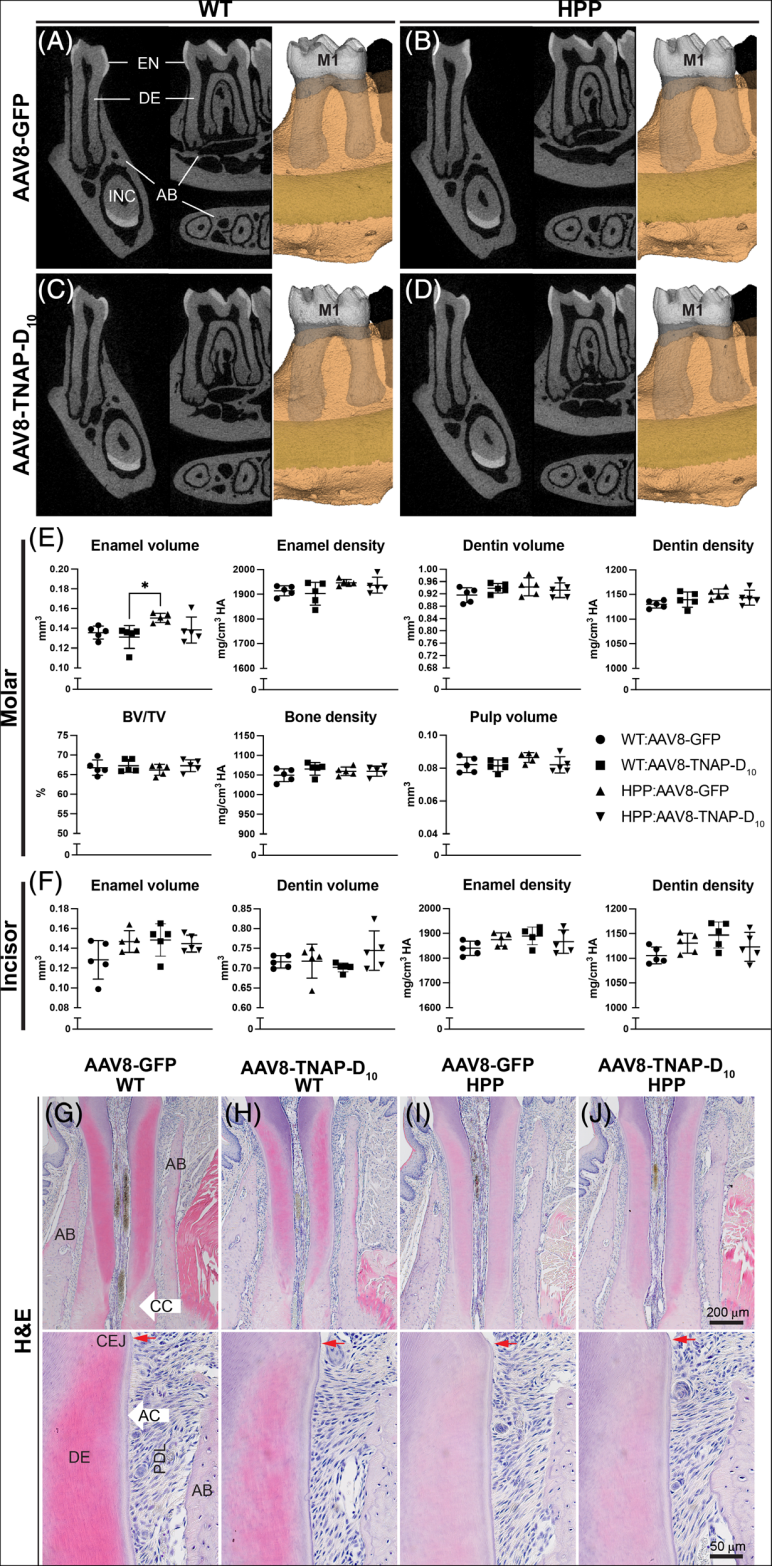
Micro‐CT analysis of dentoalveolar complex from adult hypophosphatasia (HPP) mice and wild‐type (WT) littermates. 2D and 3D micro‐CT images of (*A*–*D*) first molar (M1) and incisor (INC) from AAV8‐TNAP‐D_10_‐ or AAV8‐GFP‐treated mice 60 days after injection. (*E*) Quantification of molar enamel, dentin, alveolar bone, and pulp parameters. (*F*) Quantification of incisor enamel and dentin volumes and densities. (*G*–*J*) Hematoxylin and eosin (H&E) staining of tissue organization of teeth. No obvious difference in tissue organization, periodontal attachments, or acellular cementum between AAV8‐TNAP‐D_10_‐ or AAV8‐GFP‐treated mice. Statistical analysis was performed by one‐way ANOVA followed by Tukey's multiple comparison test. Differences were statistically significant at *p < 0.05. AB = alveolar bone; CC = cellular cementum; CEJ = cemento‐enamel junction; DE = dentin; EN = enamel; PDL = periodontal ligaments.

**Fig. 8:**
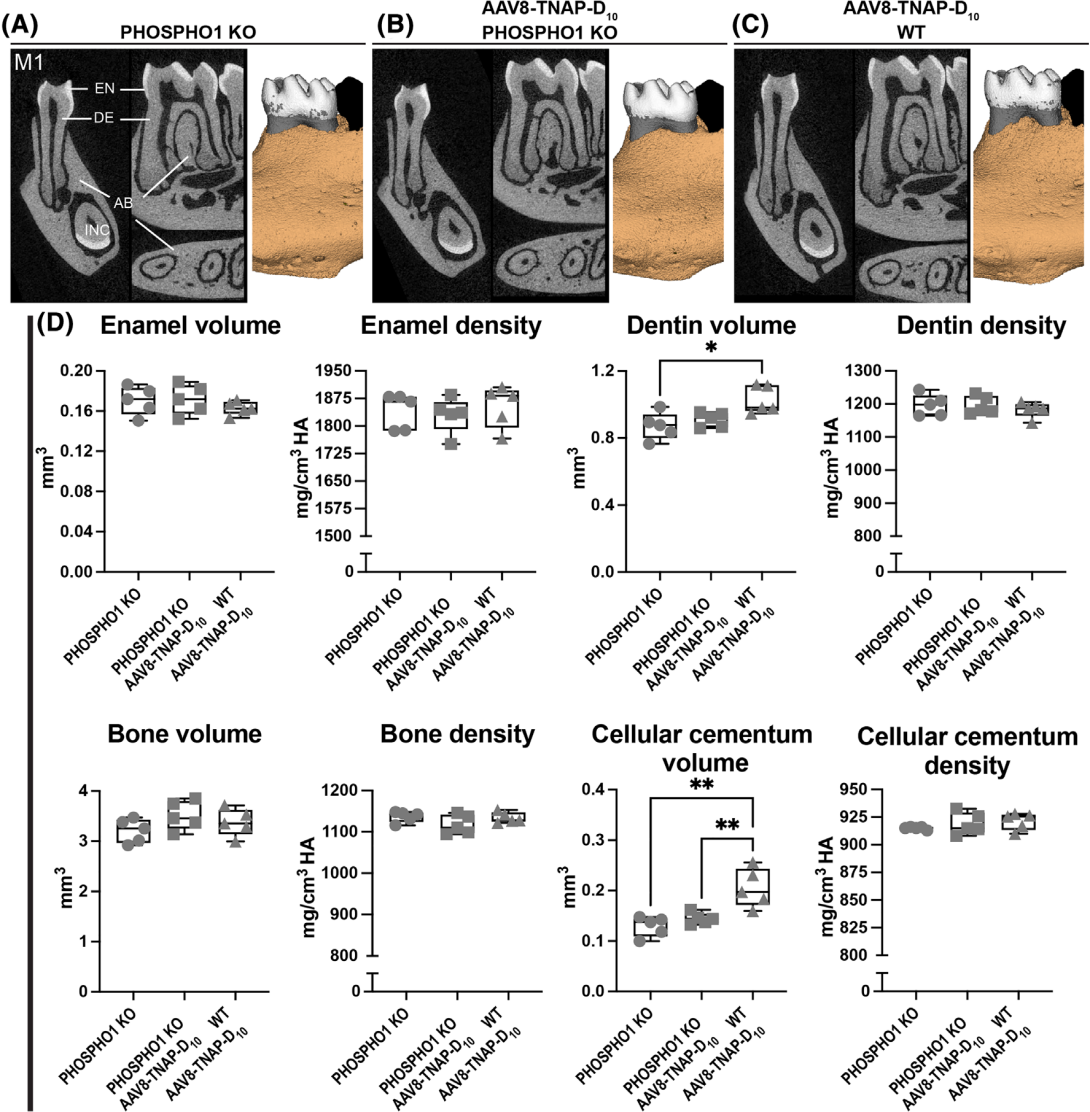
Micro‐CT analysis of dentoalveolar complex from *Phospho1* knockout (KO) and wild‐type (WT) littermates. 2D and 3D micro‐CT images of (*A*–*C*) first molar (M1) and incisor (INC) from AAV8‐TNAP‐D_10_‐ or AAV8‐GFP‐treated mice 90 days after injection. (*D*) Quantification of molar enamel, dentin, alveolar bone, and cellular cementum parameters. No obvious difference in tissue organization, or periodontal attachments, except for higher dentin and acellular cementum volumes from AAV8‐TNAP‐D_10_‐treated WT mice when compared with *Phospho1* KO mice treated with AAV8‐TNAP‐D_10_ and AAV8‐GFP. Statistical analysis was performed by one‐way ANOVA followed by Tukey's multiple comparison test. Differences were statistically significant at *p < 0.05 and **p < 0.01. AB = alveolar bone; DE = dentin; EN = enamel.

Micro‐CT analysis of the first mandibular molar and associated alveolar bone showed that adult HPP mice had no evident defects in enamel, dentin/cementum, bone volumes, and mineral densities compared with untreated (AAV8‐GFP) WT or AAV8‐TNAP‐D_10_‐treated WT and HPP mice (Fig. 7*A–E*). However, there was a statistically significant difference in the enamel volume between AAV8‐TNAP‐D_10_‐treated WT and AAV8‐GFP HPP (Fig. 7*E*). Similarly, micro‐CT analysis of continuously erupting incisors showed no significant difference in enamel and dentin volumes and densities (Fig. 7*A–D*, *F*). Additionally, AAV8‐TNAP‐D_10_‐mediated gene therapy for 60 days had no adverse effects as no difference was observed between treated and untreated WT teeth (Fig. 7*A*, *C*). Histological analysis of untreated HPP teeth revealed no morphological differences compared with untreated WT or both AAV8‐treated HPP and WT littermates. There was no obvious difference in tissue organization, periodontal attachments, or acellular cementum between treated and AAV8‐GFP HPP (Fig. 7*G–J*). Similar results were found in the dentoalveolar complex of *Phospho1* ^−/−^ mice, showing milder mineralization defects. There were no evident phenotypic changes except for an increase in the cellular cementum volume in AAV8‐TNAP‐D_10_‐treated WT mice when compared with *Phospho1* ^*−/−*^‐treated and untreated mice (Fig. 8*A–D*).

## Discussion

Diverse therapeutic methods have been proposed for the treatment of HPP, such as bone marrow transplantation,^(^ ^38^, ^39^, ^40^ ^)^ growth hormone therapy in infants,^(^ ^41^, ^42^ ^)^ and the use of parathyroid hormone (PTH)^(^ ^43^, ^44^ ^)^ and anti‐sclerostin antibodies.^(^ ^45^, ^46^ ^)^ The mechanism of action of PTH is based on stimulation of bone formation,^(^ ^47^ ^)^ decreasing pain and pseudofractures or fractures, and improving biochemical and densitometric parameters.^(^ ^43^, ^48^, ^49^ ^)^ Increased levels of ALP activity, as well as decreased levels of pyridoxal‐5′‐phosphate, one of the TNAP substrates, and an increase of lumbar bone mineral density (BMD) were observed with teriparatide treatment, a synthetic recombinant amino acid fragment of human PTH. However, the literature indicates that the response to this drug may vary depending on the TNAP mutation.^(^ ^50^, ^51^, ^52^ ^)^ Monoclonal anti‐sclerostin antibody treatment has been demonstrated to reduce bone resorption and stimulate bone formation, increasing the BMD in HPP patients.^(^ ^45^ ^)^ Bisphosphonates and calcium and/or vitamin D3 have been used to treat HPP; however, bisphosphonate treatment in adult HPP may increase the risk of fracture,^(^ ^53^, ^54^, ^55^ ^)^ as these are analogs of PP_i_ and therefore contribute to the inhibition of HA formation. Calcium and/or vitamin D3 supplementation is not recommended for HPP patients with normal calcium levels because it could cause hypercalcemia, hyperphosphatemia, and hypercalciuria.^(^ ^56^ ^)^

Several early attempts of ERT for HPP, such as the use of the transfusion of blood plasma rich in TNAP or purified human liver or placental alkaline phosphatase,^(^ ^57^, ^58^ ^)^ were unsuccessful to rescue the severe forms of HPP, suggesting that probably TNAP would need to be targeted to mineralizing tissues to be an effective treatment. Asfotase alfa, a mineral‐targeted form of recombinant TNAP, was approved for the treatment of pediatric‐onset HPP in 2015 after successful preclinical trials showing a significant improvement in skeletal mineralization and prevention of seizures in a model of severe infantile HPP^(^ ^18^, ^19^, ^20^, ^21^, ^22^ ^)^ and clinical trials in pediatric patients with perinatal/infantile HPP.^(^ ^23^ ^)^ Although ERT using asfotase alfa markedly improves the life span, skeletal phenotype, motor function, and quality of life in patients with pediatric‐onset HPP, limitations of this treatment exist, and they are: (i) the requirement for lifelong treatment with up to six injections per week that can cause severe injection site reactions^(^ ^59^ ^)^; (ii) extremely high cost of the biologic; and (iii) inaccessibility of this treatment for patients with the adult forms of the disease who are left with only traditional palliative management of their orthopedic and dental issues.

Alternative options for delivery of the therapeutic biologic, such as viral vector‐mediated ERT, have shown efficacy in preserving life and improving the skeletal phenotype in the mouse model of infantile HPP.^(^ ^24^, ^25^, ^26^, ^27^, ^28^, ^29^, ^30^ ^)^ Two recent articles described that a single dose of AAV8‐TNAP‐D_10_ led to survival and prevention of the bone and dentoalveolar phenotype in a severe infantile HPP mouse model.^(^ ^28^, ^30^ ^)^ In this present study, we demonstrate that a single intramuscular injection of this same AAV8‐TNAP‐D_10_ vector improved the long bone phenotype of a model of late‐onset adult HPP 60 days after administration of the viral vector, as visualized by radiographical images, and detailed μCT and histology. Radiography and histology also showed typical development of the cranium, teeth, and correlated periodontal tissues in treated adult HPP mice compared with WT mice. Unlike *Alpl* ^*−/−*^, a model of infantile HPP null for TNAP activity that displays severe skeletal and dental defects throughout, the *Alp* ^*Prx1/Prx1*^ model of adult HPP presented changes mainly in the long bones. This difference is likely explained by the expression pattern of the *Prx1* promoter that targets Cre expression to osteoprogenitors and their progeny in the limb mesenchyme,^(^ ^60^ ^)^ thus leading to a more severe phenotype in the appendicular than in the axial skeleton.

In 2015, we demonstrated that the genetic upregulation of TNAP activity in vascular smooth muscle (*Tagln‐Cre*; *Hprt* ^*ALPL/Y*^) cells leads to severe medial vascular calcification, hypertension, cardiac hypertrophy, and death^(^ ^61^ ^)^ and that the pan‐endothelial overexpression of TNAP (*Tie2‐Cre*; *Hprt* ^*ALPL/Y*^) induces systemic arterial calcification that leads to severe atherosclerosis and accelerated mortality when combined with an atherogenic diet.^(^ ^62^ ^)^ Given that TNAP‐D_10_, similarly to asfotase alfa, has a targeting motif designed to specifically bind to sites of mineralization, it is inescapable that it also has the potential to bind to sites of ectopic calcification, where it may promote vascular calcification and adverse cardiovascular consequences. Given that HPP patients undergoing lifelong treatment with asfotase alfa may develop comorbidities associated with vascular calcification, such as diabetes or chronic kidney disease‐mineral bone disorders (CKD‐MBD), evaluating the consequences of such binding may be clinically relevant. In this study, CKD was induced in adult HPP mice,^(^ ^36^ ^)^ causing severe vascular calcification within 8 weeks. Calcification was observed in the kidneys, hearts, and aortas of both AAV8‐GFP control‐treated and AAV8‐TNAP‐D_10_‐treated female WT and adult HPP mice. Interestingly, ectopic calcification was found as small calcification loci in WT mice, whereas in the late‐onset HPP mouse model, calcification deposits were large, especially in the heart and aorta. This is a short observational window of only 60 days, and future experiments will be designed to look for the effects of CKD‐MBD on animals after lifelong treatment with mineral‐targeted TNAP.

Importantly, sex differences were identified in our study. The CAG promoter was used to generate the recombinant type 8 AAV vector expressing TNAP‐D_10_, and it seems to sensitively respond to hormones. Sex hormones influence liver expression in CAG.^(^ ^63^ ^)^ A study showed that when using CAG promoter‐driven lacZ in transgenic rats, the sexual dimorphism of transgene expression occurred mainly in the liver and that testosterone was a factor to mediate the upregulation of the reporter gene in the transgenic rat. The regulatory mechanism of sex‐different transgene expression patterns in the liver has not been well comprehended. The hypothesis is that it could be due to a specific epigenetic regulation. Therefore, the underlying mechanism of this transgene expression should be further examined.^(^ ^63^ ^)^

PHOSPHO1 is a matrix vesicle (MV)‐associated enzyme critically important for the mechanically competent mineralization of the skeleton and the avoidance of spontaneous fractures.^(^ ^19^, ^64^ ^)^ Ablation of the *Phospho1* gene leads to osteomalacia, fractures, early‐onset scoliosis, and defective MV biogenesis.^(^ ^19^, ^65^ ^)^ Mice with a *Phospho1* gene ablation live to adulthood but develop scoliosis starting from birth, osteomalacia, and greenstick fractures, accompanied by the elevation in plasma PP_i_ and a decrease in plasma TNAP activity.^(^ ^13^, ^19^, ^64^, ^66^ ^)^ Loss of *Phospho1* also causes mineralization defects in dentin and alveolar bone.^(^ ^67^ ^)^ Importantly, the simultaneous deletion of both the *Alpl* and *Phospho1* genes leads to embryonic lethality with a complete absence of skeletal and dental mineralization.^(^ ^19^, ^68^ ^)^

Given that the symptomatology of *Phospho1* ^*−/−*^ mice resembles many of the manifestations of HPP, we surmised that *Phospho1* gene mutations could be associated with cases of pseudo‐HPP, where TNAP may be normal to subnormal, but an *ALPL* mutation has not been identified.^(^ ^11^ ^)^ Overexpressing TNAP under control of the *ApoE* promoter, in the *Phospho1* ^*−/−*^ mice did not prevent the skeletal defect in that model but ablating *Spp1* (osteopontin [OPN]) in the *Phospho1* ^*−/−*^ mice helped improve the skeletal phenotype.^(^ ^69^ ^)^ We concluded that although *Alpl* and *Phospho1* deficiencies lead to similar skeletal phenotypes and a parallel increase in the plasma levels of PP_i_ and OPN, there was an apparent dissociation in the hierarchical roles of these potent inhibitors of mineralization, with elevated PP_i_ and elevated OPN levels causing the respective skeletal phenotypes in *Alpl* ^*−/−*^ and *Phospho1* ^*−/−*^ mice. However, when we published those studies, we had not yet discovered that the *Phospho1* deficiency also caused a drastic decrease in the number of MVs produced by chondrocytes and osteoblasts,^(^ ^65^ ^)^ which likely prevented the *ApoE‐tnap*‐expressing cells from delivering TNAP‐loaded MVs to the extracellular matrix (ECM) to normalize PP_i_ and OPN concentrations. With this new insight into the pathophysiology of the PHOSPHO1 deficiency, we surmised that treating *Phospho1* ^*−/−*^ mice with mineral‐targeted TNAP, which targets the ECM directly, might lead to a therapeutic benefit, not unlike that experienced by the *Alpl* ^*−/−*^ *and Alpl* ^*Prx1/Prx1*^ models of HPP.

Here we show that, indeed, the use of AAV8‐TNAP‐D_10_ prevents severe scoliosis that characterizes this murine model with improvements in the trabecular bone and a reduced marrow space in the vertebrae of AAV8‐TNAP‐D_10_‐treated *Phospho1* ^*−/−*^ mice. In a recent perspective,^(^ ^11^ ^)^ we discussed that *Phospho1* gene mutations may underlie some cases of “pseudo‐HPP,” where ALP may be normal to subnormal, but *ALPL* mutation(s) have not been identified. These patients would be expected to have skeletal defects such as osteomalacia and fractures consistent with HPP but featuring dental phenotypes with mild enamel and dentin effects and intact cementum, periodontal attachment, and no premature tooth loss. The present data, treating mice with a *Phospho1*‐deficient pseudo‐HPP phenotype, show efficacy in the skeletal manifestations (complete prevention of scoliosis and improvement in the trabecular architecture), while there were no dentoalveolar deficits before or after treatment. In addition, this result suggests that the delivery of TNAP corrects the skeletal defects in the *Phospho1* ^*−/−*^ mice by normalizing the TNAP levels, which are low in Phospho1 KO mice, and not by substituting for the absence of PHOSPHO1 per se.

In summary, a single dose of an AAV8 vector encoding TNAP‐D_10_ improved the bone phenotype of long bones on adult HPP mice without apparent issues in soft organs after short‐term treatment, eliminating the requirement for lifelong treatment with up to six injections per week of mineral‐targeted that often lead to severe injection site reactions. In addition, this treatment corrected congenital scoliosis in the model of pseudo‐HPP caused by *Phospho1* gene deletion. Thus, viral‐mediated ERT using AAV8‐TNAP‐D_10_ shows promise as a therapeutic approach in adult patients with heritable forms of osteomalacia.

## Conflicts of Interest

JLM is a consultant for Aruvant. JLM and KM are co‐inventors of a patent application for the use of viral‐mediated administration of mineral‐targeted TNAP for the treatment of HPP and pseudo‐HPP. FAO, FFM, YK, SN, CF, and BLF state that they have no conflicts of interest.

## Author Contributions

**Flavia Amadeu de Oliveira:** Conceptualization; data curation; formal analysis; investigation; methodology; validation; visualization; writing – original draft; writing – review and editing. **Fatma F. Mohamed:** Conceptualization; data curation; formal analysis; methodology; validation; writing – review and editing. **Yuka Kinoshita:** Conceptualization; writing – review and editing. **Sonoko Narisawa:** Conceptualization; writing – review and editing. **Colin Farquharson:** Conceptualization; writing – review and editing. **Koichi Miyake:** Conceptualization; resources; writing – review and editing. **Brian L Foster:** Conceptualization; data curation; writing – review and editing. **Jose Luis Millan:** Conceptualization; funding acquisition; resources; supervision; writing – original draft; writing – review and editing.

## Supporting information


**Supplemental Table S1.** Biochemical Analysis of Late‐Onset HPP and WT Treated Mice Before Treatment
**Supplemental Table S2.** Biochemical Analysis of Late‐Onset HPP and WT Treated Mice
**Supplemental Table S3.** Biochemical Analysis of *Phospho*1 KO and WT Treated Mice

**Supplemental Fig. S1.** Biochemical analysis in serum/plasma from 2‐month‐old females and males adult HPP mice and WT siblings before injection. (*A*, *B*) Serum alkaline phosphatase activity. (*C*, *D*) Plasma PPi levels. (*E*, *F*) Serum calcium assay. (*G*, *H*) Serum phosphorus concentration. (*I*, *J*) Blood urea nitrogen (BUN) levels in serum. Statistical analysis was performed by unpaired *t* test. *p < 0.05. **p < 0.01. ****p < 0.0001.

**Supplemental Fig. S2.** Radiographical findings of female adult HPP mice bone phenotype. Radiographic images of whole skeletal tissue, with higher magnification of skull along with spine (2×), vertebra (2×), and hemimandibles (3×). (*A*, *B*) Females WT treated with control AAV8‐GFP and AAV8‐TNAP‐D_10_. (*C*, *D*) Females adult HPP AAV8‐GFP or AAV8‐TNAP‐D10 treated mice after 60 days of injection.

**Supplemental Fig. S3.** Radiographical findings of males adult HPP mice bone phenotype. Radiographic images of whole skeletal tissue, with higher magnification of skull along with spine (2×), vertebra (2×), and hemimandibles (3×). (*A*, *B*) Males WT treated with vehicle AAV8‐GFP and AAV8‐TNAP‐D10. (*C*, *D*) Males adult HPP AAV8‐GFP or AAV8‐TNAP‐D10 treated mice after 60 days of injection.

**Supplemental Fig. S4.** Radiographical findings of female *Phospho1* KO mice bone phenotype. Radiographic images of whole skeletal tissue, with higher magnification of skull along with spine (2×), head (4×), hemimandibles (3×), vertebra (2×), and long bones (2×). Females *Phospho1* KO treated with control (*A*) AAV8‐GFP or (*B*) AAV8‐TNAP‐D_10_ after 90 days of injection.

**Supplemental Fig. S5.** Radiographical findings of male *Phospho1* KO mice bone phenotype. Radiographic images of whole skeletal tissue, with higher magnification of skull along with spine (2×), head (4×), hemimandibles (3×), vertebra (2×), and long bones (2×). Males *Phospho1* KO treated with control (*A*) AAV8‐GFP or (*B*) AAV8‐TNAP‐D_10_ after 90 days of injection.

**Supplemental Fig. S6.** Alizarin red staining of soft organs from adult HPP and WT mice. (*A*) Female and (*B*) male treated with AAV8‐GFP or AAV8‐TNAP‐D_10_. No evidence of ectopic calcifications was found after 60 days of vector encoding TNAP injection. First row: kidney; second row: liver; third row: heart; fourth row: aorta (20× magnification).

**Supplemental Fig. S7.** Representative images of ectopic calcification on soft organs from adult females HPP and WT mice under CKD diet. Alizarin red staining was performed to show the ectopic calcification in soft organs and vasculature in late‐onset HPP mouse model and WT littermates as control. Histological sections of kidney, heart, and aorta for the following experimental groups (*A*, *B*, *E*, *F*, *I*, *J*) WT mice treated with control AAV8‐GFP or AAV8‐TNAP‐D_10_, and (*C*, *D*, *G*, *H*, *K*, *L*) adult HPP injected mice with AAV8‐GFP or AAV8‐TNAP‐D_10_. Kidney: upper panels 996 uM, lower panels 100 uM magnification; heart: upper panels 996 uM, lower panels 50 uM magnification; aorta: upper panels 100 uM, lower panels 50 uM magnification. (*M*) Alizarin red S quantification.
