# Supplementary material for: Gene Therapy Using Recombinant AAV Type 8 Vector Encoding TNAP‐D10 Improves the Skeletal Phenotypes in Murine Models of Osteomalacia
Source: JBMR Plus. 2022 Dec 15;7(1):e10709. doi: 10.1002/jbm4.10709 (PMC9850441; doi:10.1002/jbm4.10709)
Supplement: Supplementary file 1 — Supplemental Table S1. Biochemical Analysis of Late‐Onset HPP and WT Treated Mice Before Treatment Supplemental Table S2. Biochemical Analysis of Late‐Onset HPP and WT Treated Mice Supplemental Table S3. Biochemical Analysis of Phospho1 KO and WT Treated Mice [file JBM4-7-e10709-s003.docx]

**Supplemental material**

**Suppl. Figure 1**- Biochemical analysis in serum/plasma from 2 months old females and males adult HPP mice and WT siblings prior to injection. (A-B) Serum alkaline phosphatase activity. (C-D) Plasma PPi levels. (E-F) Serum calcium assay. (G-H) Serum phosphorus concentration. (I-J) Blood urea nitrogen (BUN) levels in serum. Statistical analysis was performed by Unpaired t-test. *P<0.05. **P<0.01. ****P<0.0001.

**Suppl. Table 1**. Biochemical analysis of late-onset HPP and WT treated mice before treatment.

|  |  | **Prior to treatment** | | ANOVA |
| --- | --- | --- | --- | --- |
| Biochemical markers |  | WT | HPP | P-value |
| ALP, ng/mL |  | F: 45.95±4.24 | F: 22.77±3.14 | <0.0001^****^ |
|  |  | M: 42.36±4.28 | M: 22.35±3.58 | <0.0001^****^ |
| PP_i_, µM |  | F: 0.262±0.15 | F: 0.828±0.40 | 0.0068^**^ |
|  |  | M: 0.273±0.16 | M: 0.798±0.22 | <0.0001^****^ |
| Ca, mg/dL |  | F: 12.73±0.84 | F: 12.25±0.69 | 0.2402^ns^ |
|  |  | M: 12.67±1.22 | M: 12.64±0.81 | 0.9499^ns^ |
| P, mg/dL |  | F: 11.24±2.45 | F: 7.14±3.87 | 0.0394^*^ |
|  |  | M: 8.47±3.35 | M: 7.92±2.59 | 0.6993^ns^ |
| BUN, mg/dL |  | F: 47.11±7.98 | F: 49.74±7.89 | 0.5385^ns^ |
|  |  | M: 54.35±5.69 | M: 50.26±6.11 | 0.1645^ns^ |

Statistical analysis was performed by Unpaired t test. *P<0.05. **P<0.01. ****P<0.0001. ^ns^P>0.05 - Not significant. Comparison: WT vs. HPP prior to the treatment. F- females; M- males.

|  |  | Genotype and treatment | | | | ANOVA |
| --- | --- | --- | --- | --- | --- | --- |
| Biochemical markers |  | WT  AAV8-GFP | WT  AAV8-TNAP-D_10_ | HPP  AAV8-GFP | HPP  AAV8-TNAP-D_10_ | P-value |
| ALP, ng/mL |  | F:44.67±7.37 | F:2482.00±1030.00 ^a^ | F:24.97±4.62 | F:214260.00±23307.00 ^b^ | <0.0001 |
|  |  | M:31.53±1.55 | M:469950.00±52957^a^ | M:20.91±2.62 | M:484693.00±155911.00^bd^ | <0.0001 |
| PP_i_, µM |  | F:0.243±0.05 | F:0.104±0.05 | F:0.752±0.10^c^ | F:0.530±0.14^d^ | <0.001 |
|  |  | M:0.171±0.10 | M:0.072±0.03 | M:0.534±0.13^c^ | M:0.113±0.05 ^b^ | <0.001 |
| Ca, mg/dL |  | F:11.30±1.08 | F:14.02±1.94^a^ | F:11.52±0.39 | F:11.42±0.47^d^ | <0.05 |
|  |  | M:11.34±2.00 | M:12.46±0.57 | M:12.85±1.70 | M:12.39±0.75 | ns |
| P, mg/dL |  | F:4.57±0.61 | F:5.73±0.97 | F:12.22±0.89^c^ | F:11.56±2.72^d^ | <0.01 |
|  |  | M:3.85±1.05 | M:5.12±1.18 | M:8.47±1.84^c^ | M:7.94±1.37^d^ | <0.05 |
| BUN, mg/dL |  | F:54.02±9.69 | F:59.23±15.69 | F:48.62±4.37 | F:54.69±3.82 | ns |
|  |  | M:50.11±5.58 | M:60.63±12.95 | M:56.21±9.32 | M:53.64±5.14 | ns |

**Suppl. Table 2**: Biochemical analysis of late-onset HPP and WT treated mice.

Statistical analysis was performed by One-way ANOVA followed by Tukey’s multiple comparison test. *P<0.05. **P<0.01. ***P<0.001. ****P<0.0001. ^ns^P>0.05 - Not significant. Comparisons: a) WT: GFP vs. TNAP; b) HPP: GFP vs. TNAP; c) GFP: WT vs. HPP; d) TNAP: WT vs. HPP. F- females; M- males.

|  |  | Genotype and Treatment | | | ANOVA |
| --- | --- | --- | --- | --- | --- |
| Biochemical markers |  | WT  AAV8-TNAP-D_10_ | PHOSPHO1 KO  AAV8-TNAP-D_10_ | PHOSPHO1 KO  AAV8-GFP | P-value |
| ALP, ng/mL |  | 45d-F: 51122±12212  90d-F: 38762±8833 | 45d-F: 54101±1807 ^a^  90d-F: 54027±16032^a^ | 45d-F: 70.65 ±18.28  90d-F: 41.17±6.973 | <0.0001  <0.0001 |
|  |  | 45d-M: 53781±12749  90d-M: 51046±17115 | 45d-M: 93250 ±24000^ab^  90d-M: 74912±26568 | 45d-M: 77.55±20.72  90d-M: 36.56±7.307 | <0.05  <0.01 |
| PP_i_, µM |  | 45d-F: 0.08267±0.01168  90d-F: 0.1273±0.124 | 45d-F: 0.048±0.02234  90d-F: 0.05167±0.04193^a^ | 45d-F: 0.0756±0.06438  90d-F: 0.3964±0.1853 | ns  <0.05 |
|  |  | 45d-M: 53781±12749  90d-M: 51046±17115 | 45d-M: 93250 ±24000^ab^  90d-M: 74912±26568 | 45d-M: 77.55±20.72  90d-M: 36.56±7.307 | <0.001 |
| Ca, mg/dL |  | 45d-F: 13.02±0.8675  90d-F: 10.71±1.001 | 45d-F: 12.47±0.6672  90d-F: 13.94±0.1407 ^ab^ | 45d-F: 12.51±0.828  90d-F: 11.13±0.8477 | ns  <0.05 |
|  |  | 45d-M: 13.01±0.5232  90d-M: 12.3±1.584 | 45d-M: 12.94 ±0.8042  90d-M: 11.91±0.889 | 45d-M: 12.67±0.8406  90d-M: 12.06±1.207 | ns |
| P, mg/dL |  | 45d-F: 6.914±1.209  90d-F: 5.631±1.916 | 45d-F: 7.027±0.6286  90d-F: 8.115±3.359 | 45d-F: 8±1.354  90d-F: 5.878±3.089 | ns |
|  |  | 45d-M: 7.77±2.098  90d-M: 3.356±1.082 | 45d-M: 6.014±2.522  90d-M: 4.486±0.8501 | 45d-M: 8.595±2.103  90d-M: 3.162±1 | ns |
| BUN, mg/dL |  | 45d-F: 27.02±8.279  90d-F: 26.39±7.572 | 45d-F: 28.49±2.701  90d-F: 32.3±0.7988 | 45d-F: 35.01±6.459  90d-F: 33.71±10.56 | ns |
|  |  | 45d-M: 25.24±11.73  90d-M: 29.05±13.36 | 45d-M: 26.58±8.674  90d-M: 29.25±12.31 | 45d-M: 26.7±3.987  90d-M: 34.6±1.126 | ns |

**Suppl.Table 3**: Biochemical analysis of *Phospho*1 KO and WT treated mice.

Statistical analysis was performed by One-way ANOVA followed by Tukey’s multiple comparison test. *P<0.05. **P<0.01. ***P<0.001. ****P<0.0001. ^ns^P>0.05 - Not significant. Comparisons: a) *Phospho1* KO: AAV8-GFP vs. AAV8-TNAP-D_10_; b) WT vs. *Phospho1* KO AAV8-TNAP-D_10_ treated mice. F- females; M- males.

**Suppl. Figure 2** – Radiographical findings of female adult HPP mice bone phenotype. Radiographic images of whole skeletal tissue, with higher magnification of skull along with spine (2x), vertebra (2x), and hemi-mandibles (3x). (A-B) females WT treated with control AAV8-GFP and AAV8-TNAP-D_10_. (C-D) females adult HPP AAV8-GFP or AAV8-TNAP-D10 treated mice after 60 days of injection.

**Suppl. Figure 3** – Radiographical findings of males adult HPP mice bone phenotype. Radiographic images of whole skeletal tissue, with higher magnification of skull along with spine (2x), vertebra (2x), and hemi-mandibles (3x). (A-B) males WT treated with vehicle AAV8-GFP and AAV8-TNAP-D10. (C-D) males adult HPP AAV8-GFP or AAV8-TNAP-D10 treated mice after 60 days of injection.

**Suppl. Figure 4** – Radiographical findings of female *Phospho1* KO mice bone phenotype. Radiographic images of whole skeletal tissue, with higher magnification of skull along with spine (2x), head (4x), hemi-mandibles (3x), vertebra (2x), and long bones (2x). Females *Phospho1* KO treated with control (A) AAV8-GFP or (B) AAV8-TNAP-D_10_ after 90 days of injection.

**Suppl. Figure 5** – Radiographical findings of male *Phospho1* KO mice bone phenotype. Radiographic images of whole skeletal tissue, with higher magnification of skull along with spine (2x), head (4x), hemi-mandibles (3x), vertebra (2x), and long bones (2x). Males *Phospho1* KO treated with control (A) AAV8-GFP or (B) AAV8-TNAP-D_10_ after 90 days of injection.

**Suppl. Figure 6** – Alizarin red staining of soft organs from adult HPP and WT mice. (A) Female and (B) Male treated with AAV8-GFP or AAV8-TNAP-D_10_. No evidence of ectopic calcifications was found after 60 days of vector encoding TNAP injection. Upper panels: kidney. Mid panels: Heart. Lower panels: Aorta (20x mag.).

**Suppl. Figure 7** – Representative images of ectopic calcification on soft organs from adult females HPP and WT mice under CKD diet. Alizarin red staining was performed to show the ectopic calcification in soft organs and vasculature in late-onset HPP mouse model and WT littermates as control. Histological sections of kidney, heart, and aorta for the following experimental groups (A, B, E, F, I, J) WT mice treated with control AAV8-GFP or AAV8-TNAP-D_10_, and (C, D, G, H, K, L) adult HPP injected mice with AAV8-GFP or AAV8-TNAP-D_10_. Kidney: upper panels-996 uM, lower panels-100 uM mag; Heart: upper panels-996 uM, lower panels-50 uM mag; aorta: upper panels-100 uM, lower panels-50 uM mag. (M) Alizarin red S quantification.
