# Supplementary figures and images for: Gene Therapy Using Recombinant AAV Type 8 Vector Encoding TNAP‐D10 Improves the Skeletal Phenotypes in Murine Models of Osteomalacia
Source: JBMR Plus. 2022 Dec 15;7(1):e10709. doi: 10.1002/jbm4.10709 (PMC9850441; doi:10.1002/jbm4.10709)

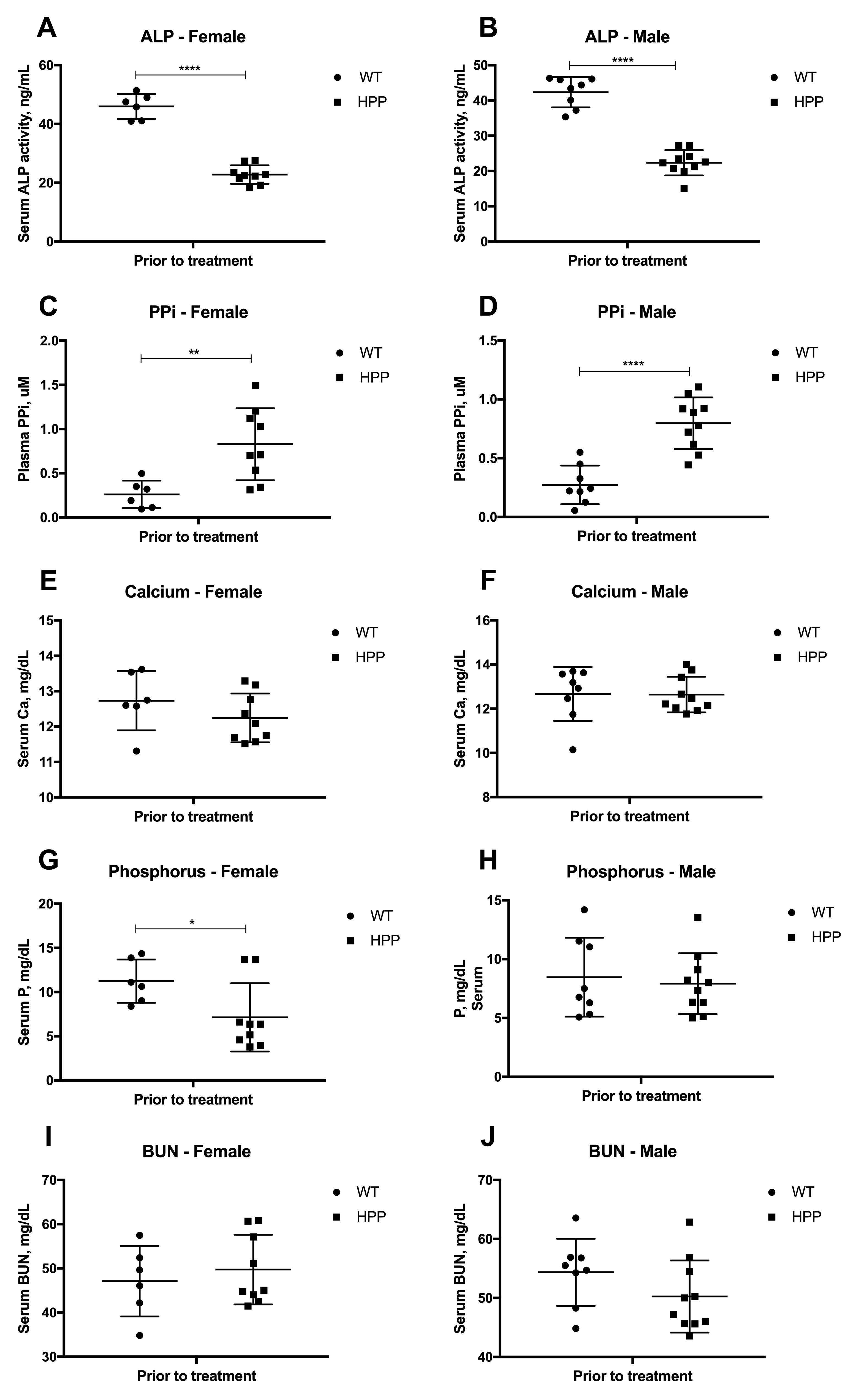

Supplement: Supplementary file 2 — Supplemental Fig. S1. Biochemical analysis in serum/plasma from 2‐month‐old females and males adult HPP mice and WT siblings before injection. (A, B) Serum alkaline phosphatase activity. (C, D) Plasma PPi levels. (E, F) Serum calcium assay. (G, H) Serum phosphorus concentration. (I, J) Blood urea nitrogen (BUN) levels in serum. Statistical analysis was performed by unpaired t test. *p < 0.05. **p < 0.01. ****p < 0.0001. [file JBM4-7-e10709-s005.tiff]

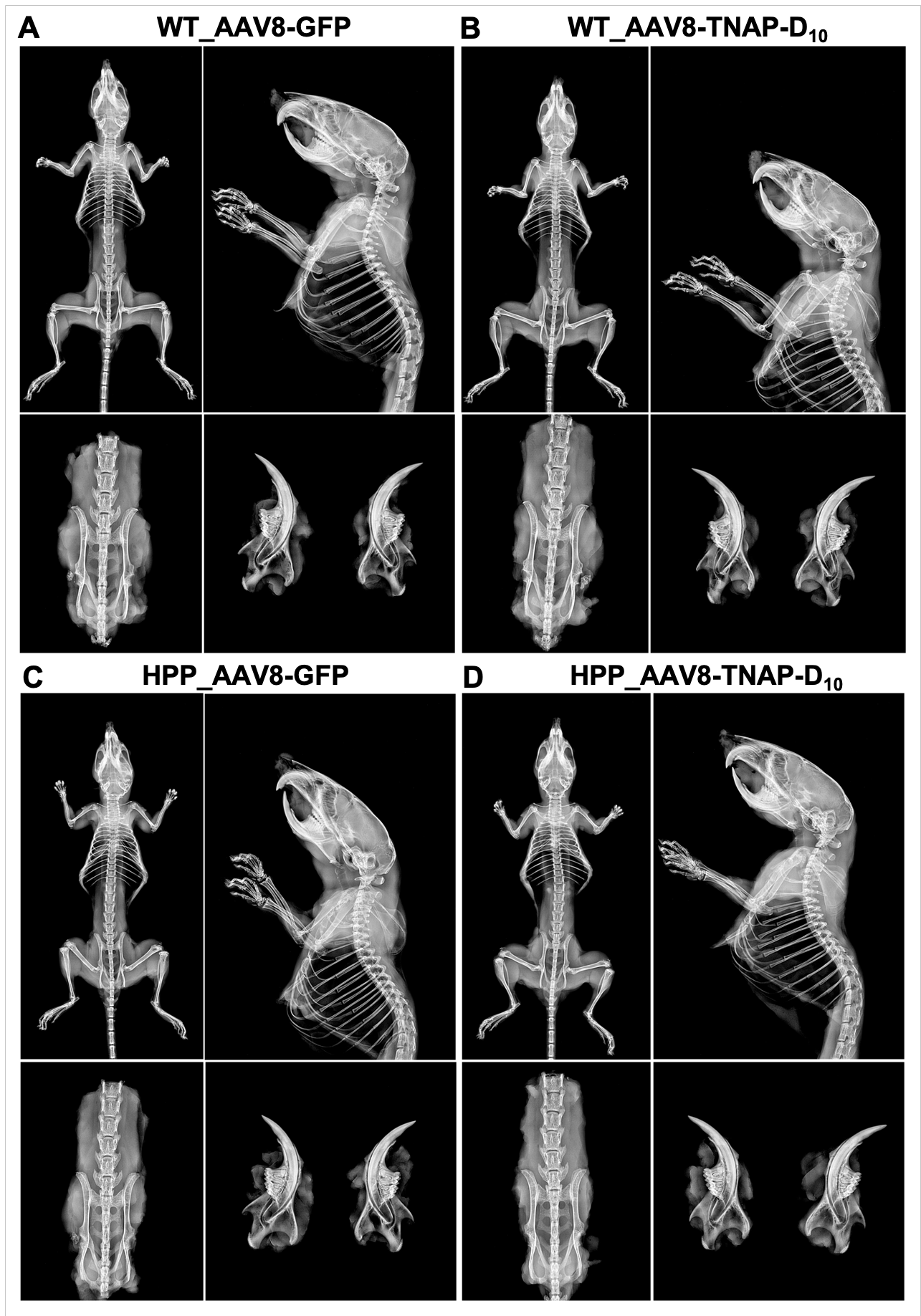

Supplement: Supplementary file 3 — Supplemental Fig. S2. Radiographical findings of female adult HPP mice bone phenotype. Radiographic images of whole skeletal tissue, with higher magnification of skull along with spine (2×), vertebra (2×), and hemimandibles (3×). (A, B) Females WT treated with control AAV8‐GFP and AAV8‐TNAP‐D10. (C, D) Females adult HPP AAV8‐GFP or AAV8‐TNAP‐D10 treated mice after 60 days of injection. [file JBM4-7-e10709-s001.tiff]

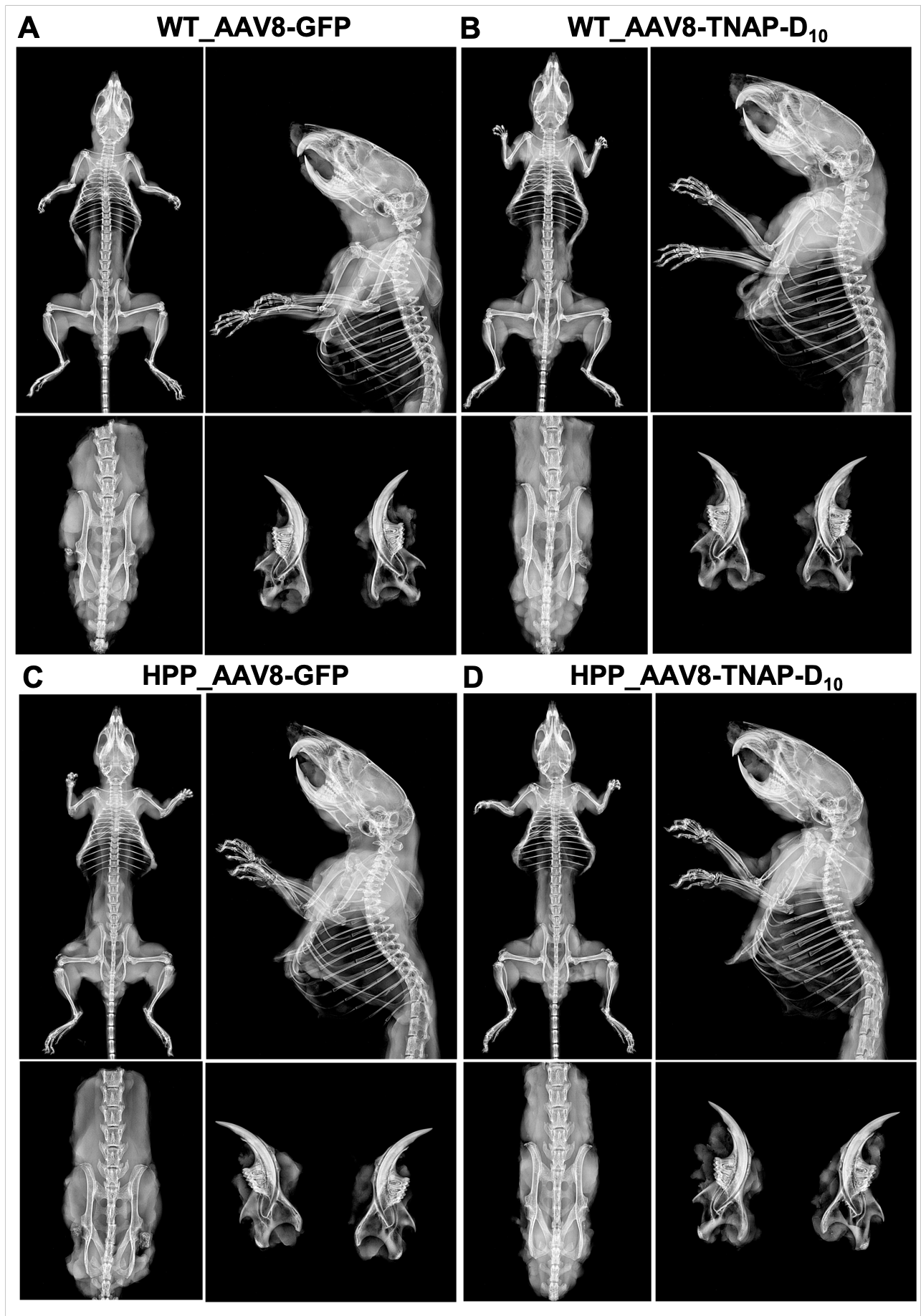

Supplement: Supplementary file 4 — Supplemental Fig. S3. Radiographical findings of males adult HPP mice bone phenotype. Radiographic images of whole skeletal tissue, with higher magnification of skull along with spine (2×), vertebra (2×), and hemimandibles (3×). (A, B) Males WT treated with vehicle AAV8‐GFP and AAV8‐TNAP‐D10. (C, D) Males adult HPP AAV8‐GFP or AAV8‐TNAP‐D10 treated mice after 60 days of injection. [file JBM4-7-e10709-s006.tiff]

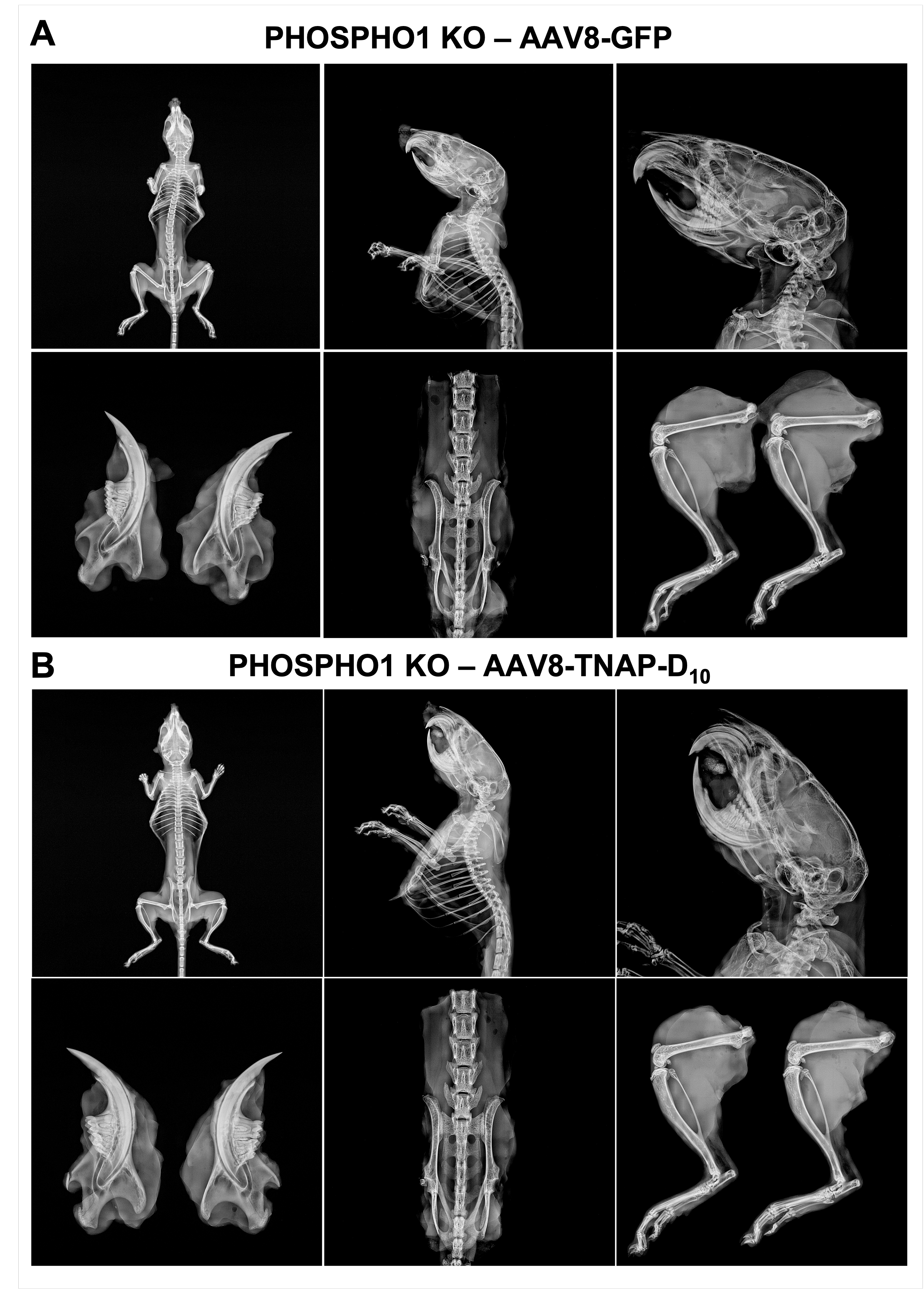

Supplement: Supplementary file 5 — Supplemental Fig. S4. Radiographical findings of female Phospho1 KO mice bone phenotype. Radiographic images of whole skeletal tissue, with higher magnification of skull along with spine (2×), head (4×), hemimandibles (3×), vertebra (2×), and long bones (2×). Females Phospho1 KO treated with control (A) AAV8‐GFP or (B) AAV8‐TNAP‐D10 after 90 days of injection. [file JBM4-7-e10709-s002.tiff]

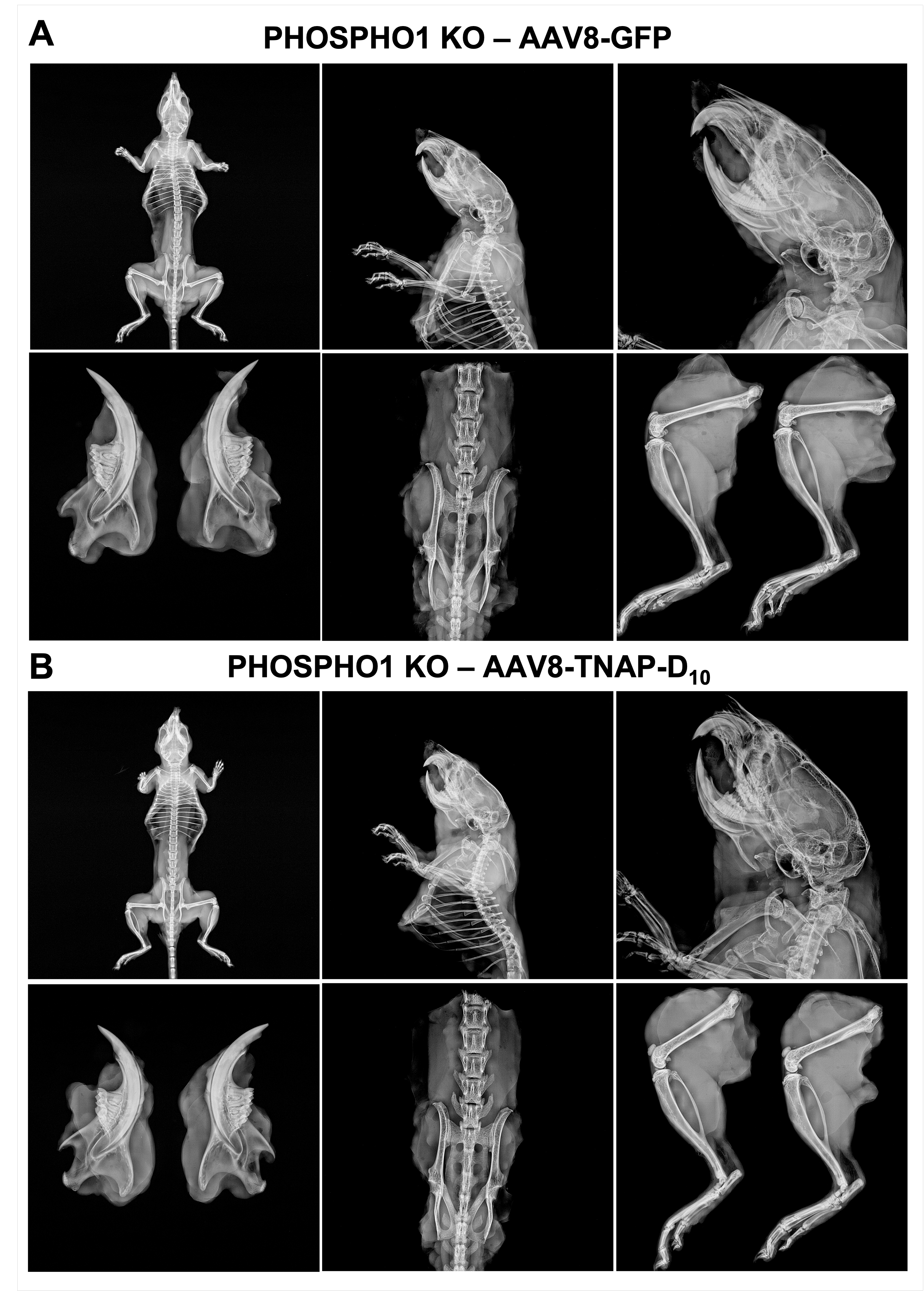

Supplement: Supplementary file 6 — Supplemental Fig. S5. Radiographical findings of male Phospho1 KO mice bone phenotype. Radiographic images of whole skeletal tissue, with higher magnification of skull along with spine (2×), head (4×), hemimandibles (3×), vertebra (2×), and long bones (2×). Males Phospho1 KO treated with control (A) AAV8‐GFP or (B) AAV8‐TNAP‐D10 after 90 days of injection. [file JBM4-7-e10709-s004.tiff]

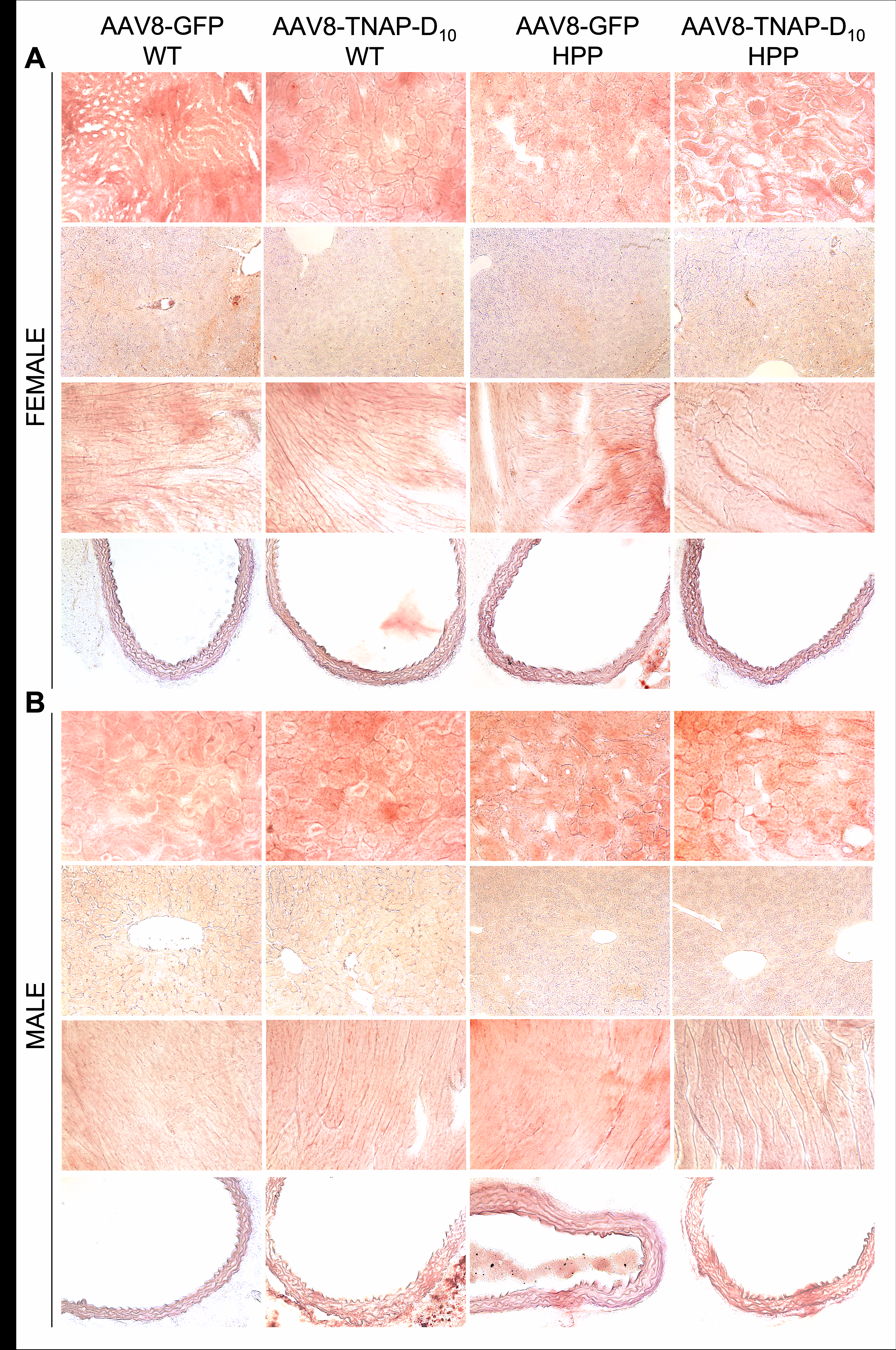

Supplement: Supplementary file 7 — Supplemental Fig. S6. Alizarin red staining of soft organs from adult HPP and WT mice. (A) Female and (B) male treated with AAV8‐GFP or AAV8‐TNAP‐D10. No evidence of ectopic calcifications was found after 60 days of vector encoding TNAP injection. First row: kidney; second row: liver; third row: heart; fourth row: aorta (20× magnification). [file JBM4-7-e10709-s007.tiff]

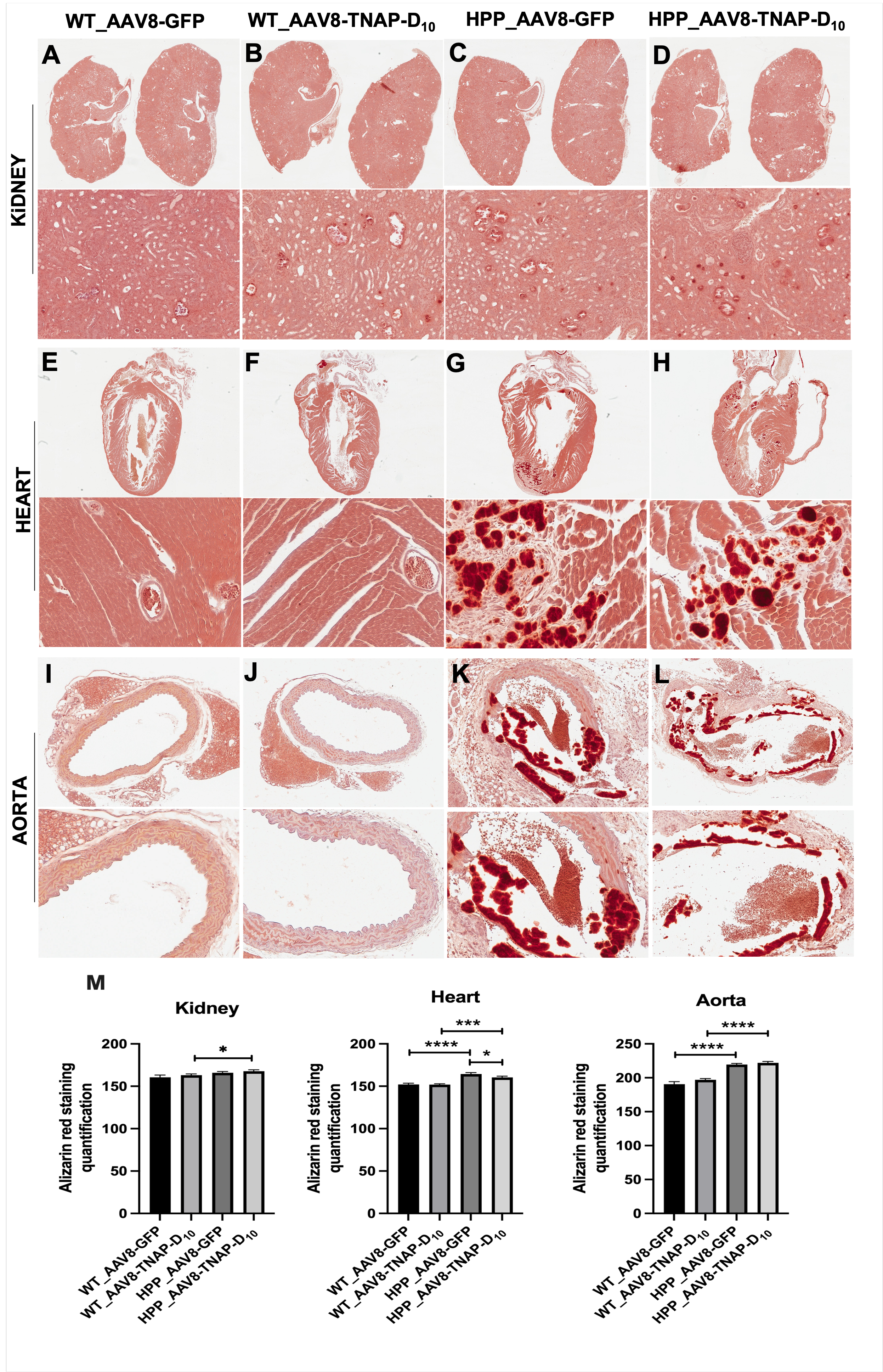

Supplement: Supplementary file 8 — Supplemental Fig. S7. Representative images of ectopic calcification on soft organs from adult females HPP and WT mice under CKD diet. Alizarin red staining was performed to show the ectopic calcification in soft organs and vasculature in late‐onset HPP mouse model and WT littermates as control. Histological sections of kidney, heart, and aorta for the following experimental groups (A, B, E, F, I, J) WT mice treated with control AAV8‐GFP or AAV8‐TNAP‐D10, and (C, D, G, H, K, L) adult HPP injected mice with AAV8‐GFP or AAV8‐TNAP‐D10. Kidney: upper panels 996 uM, lower panels 100 uM magnification; heart: upper panels 996 uM, lower panels 50 uM magnification; aorta: upper panels 100 uM, lower panels 50 uM magnification. (M) Alizarin red S quantification. [file JBM4-7-e10709-s008.tiff]
